# Payload‐Driven Design of Polymeric Carriers for Nucleic Acid Delivery: Insights from Structure–Function Relationships

**DOI:** 10.1002/advs.202512653

**Published:** 2025-11-29

**Authors:** Hanieh Moradian, Charlotte Maeve Dunne, Manfred Gossen, Matthias Hartlieb

**Affiliations:** ^1^ Institute of Active Polymers Helmholtz‐Zentrum Hereon 14513 Teltow Germany; ^2^ Berlin‐Brandenburg Center for Regenerative Therapies (BCRT) 13353 Berlin Germany; ^3^ Pantherna Therapeutics GmbH 16761 Hennigsdorf Germany; ^4^ Germany Institute of Chemistry University of Potsdam 14476 Potsdam Germany; ^5^ Fraunhofer Institute for Applied Polymer Research (IAP) D‐14476 Potsdam Germany

**Keywords:** biomaterials, biotechnology, gene delivery, nucleic acid delivery, polymeric carriers

## Abstract

Polymeric carriers are gaining increasing attention for the delivery of a range of clinically relevant nucleic acid (NA) payloads, due to their efficacy, safety, and modular chemistry. While the requirements for an “optimal” carrier depend strongly on the NA payload, research has largely centered on carrier properties rather than the unique needs of the payload. Therefore, in this review, a novel perspective inspired by protein structural organization is introduced to explore structure–function relationships of polymeric carrier systems, highlighting the diverse requirements of different NA payloads. To gain deeper mechanistic insights, the complicated polymer/NA delivery systems are deconvoluted into four hierarchical levels, including molecular composition, structural features, complexation, and 3D integration. In addition to outlining recent experimental strategies, it is emphasized that the structural diversity and tunability of polymer chemistry provide a foundation for payload‐specific carrier design. Critical advances are expected in the molecular design of polymeric carriers to enhance NA delivery efficiency, improve biological safety and incorporate multifunctionality, particularly compared to lipid‐based platforms that are currently ahead in clinical applications. A payload‐specific design framework may guide development of next‐generation polymeric carrier systems for basic research, biotechnology, and therapeutic applications.

## Graphical abstract

Polymeric carriers provide versatile platforms for nucleic acid delivery. This review introduces a novel perspective inspired by protein structural organization to explore structure–function relationships of polymeric carriers, highlighting requirements of different nucleic acid payloads. By bridging fundamental concepts with practical design strategies, a framework is provided to move beyond trial‐and‐error toward payload‐specific design, guiding next‐generation polymeric carriers for therapeutic applications.

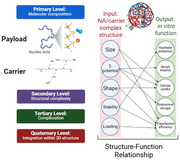

## 1 Introduction

Nucleic acid delivery has been successfully implemented across a broad range of applications in both basic science and clinical studies enabling bioengineering by the introduction, modification, or silencing of genetic material within cells^[^ ^1^ ^]^ In early gene editing studies, double‐stranded DNA (dsDNA) was utilized to correct or compensate for mutated genes associated with disease.^[^ ^2^ ^]^ As the field progressed, the use of smaller nucleic acids, such as small interfering RNAs (siRNAs) and antisense oligonucleotides enabled targeted gene silencing and regulation. The advent of synthetic messenger RNA (mRNA)‐based therapeutics, including mRNA vaccines, further expanded the possibilities by facilitating transient protein expression with improved safety profiles. More recently, CRISPR‐based genome editing has been introduced, utilizing single‐guide RNAs (sgRNAs) and often also mRNA‐encoded nucleases to achieve precise and permanent genetic modifications.^[^ ^3^ ^]^ Together, recent advances in the field of gene editing have opened new horizons for treating “undruggable” diseases by utilizing nucleic acids (NA) as bioactive entities. A concise background overview of the biological principles underlying the shuttling of genetic information into cells via NAs is provided in Section 1 of the Supporting Information.

To fulfill their intended function, all types of nucleic acids whether designed for transient or permanent effects must be efficiently delivered into cells. Given their remarkably large molecular weight, polyanionic nucleic acids are unable to passively pass through negatively charged cell membranes.^[^ ^4^ ^]^ Therefore, the transfer is accomplished using either a carrier system, or through viral vectors. Nonviral nucleic acid delivery systems, such as lipid nanoparticles (LNPs), polymeric carriers, peptide‐based vectors, inorganic nanoparticles, and nucleic acid nanomaterials, enable safe and effective cellular uptake, particularly crucial for regenerative medicine and clinical applications (Figure S2, Supporting Information). We provide an in‐depth overview of NA delivery in bioengineering and in therapeutic applications, including protein overexpression, gene knock‐down, NA vaccines, immunoengineering, and genome editing in Section 2 of the Supporting Information.

This review focuses on the rational design of polymeric carrier systems to account for diverse physicochemical requirements of different nucleic acid payloads. The first chapter provides background information regarding genetic engineering, aimed at chemists entering this field. Inspired by the four levels of protein structure, the second chapter explores the different structural levels of both nucleic acids and polymers, and their interactions in forming polyplex complexes formed between cationic polymers and negatively charged nucleic acids through electrostatic interactions. The information provided in this chapter serves as a foundation for a better understanding of structure–function relationships in the context of nucleic acid delivery. The last two chapters detail possible strategies for the rational design of polymeric carrier systems, highlighting advanced tools and additional considerations necessary for developing more effective nucleic delivery platforms. Together, these chapters aims to bridge fundamental concepts with practical design strategies, providing a framework to move beyond trial and error toward application‐tailored, mechanism‐inspired approaches for developing next‐generation polymeric carrier systems for diverse nucleic acid therapeutics.

### 1.1 Biological Barriers to Nucleic Acid Delivery with Polymeric Carriers

**Figure 1:**
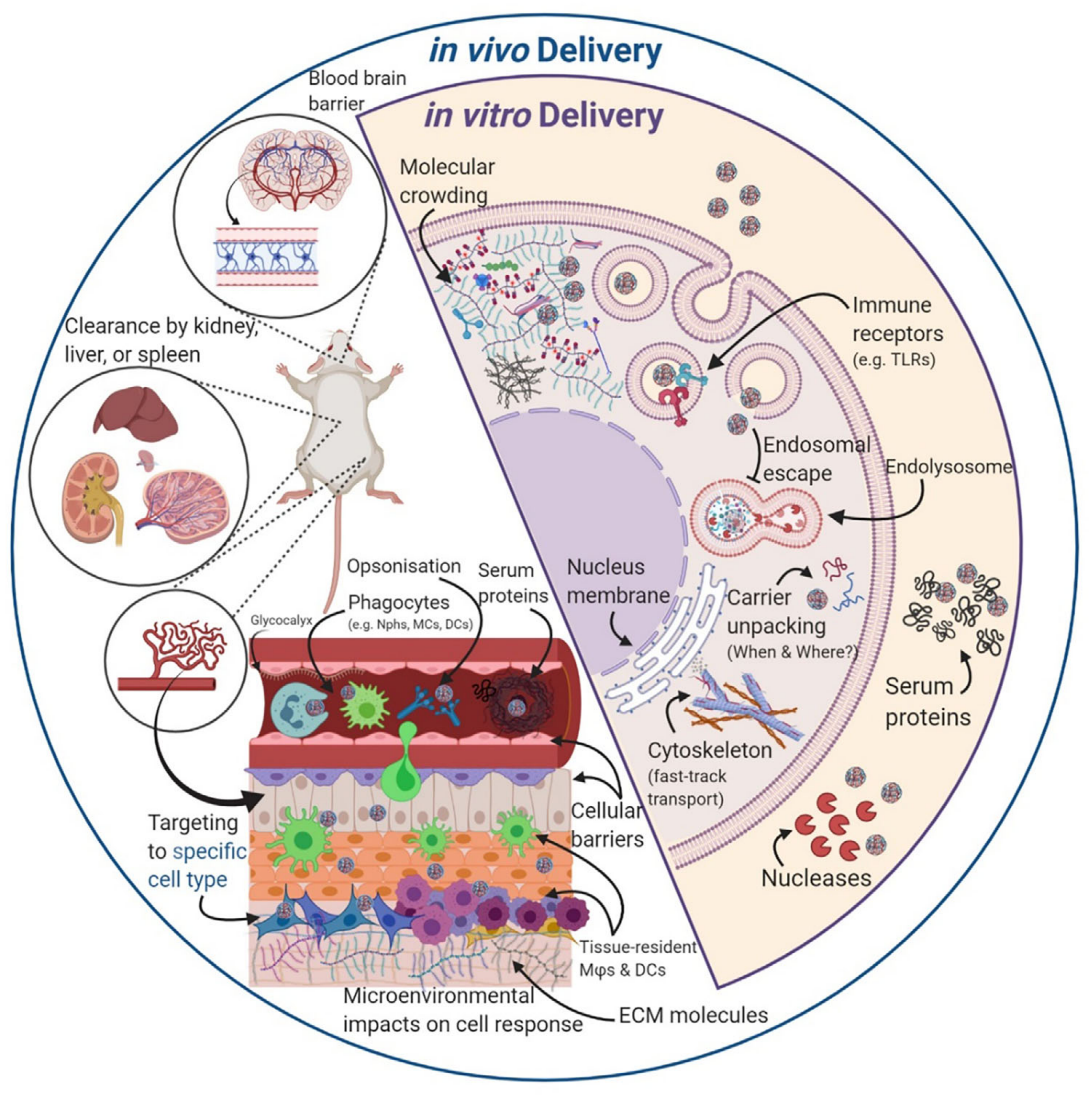
Different types of biological barriers for gene delivery corresponding to different gene delivery scenarios, namely in vitro, and in vivo. The overlap of the two circles indicates that most barriers of in vitro delivery are also valid for in vivo delivery; Nphs = neutrophils, MCs = monocytes, DCs = dendritic cells, Mφ = macrophages (Created with BioRender.com).

Nucleic acids must overcome a series of biological barriers, the nature and extent of which depend on the delivery context, whether under controlled in vitro conditions or within a more complex in vivo environment (**Figure** **1**). The following sections provide an overview of these barriers in both in vitro and in vivo contexts.

#### 1.1.1 Biological Barriers to In Vitro Nucleic Acid Delivery

The main extracellular barriers to in vitro NA delivery are degradation by nucleases and low serum stability of polyplexes. To protect the payload, a polymeric carrier should compact NAs within their chains or encapsulate them in their core.^[^ ^5^, ^6^, ^7^ ^]^ Negatively charged serum proteins can further compromise in vitro delivery, either by aggregating positively charged polyplexes or by destabilizing them through competition with polyanionic NAs.^[^ ^8^, ^9^, ^10^ ^]^

**Figure 2:**
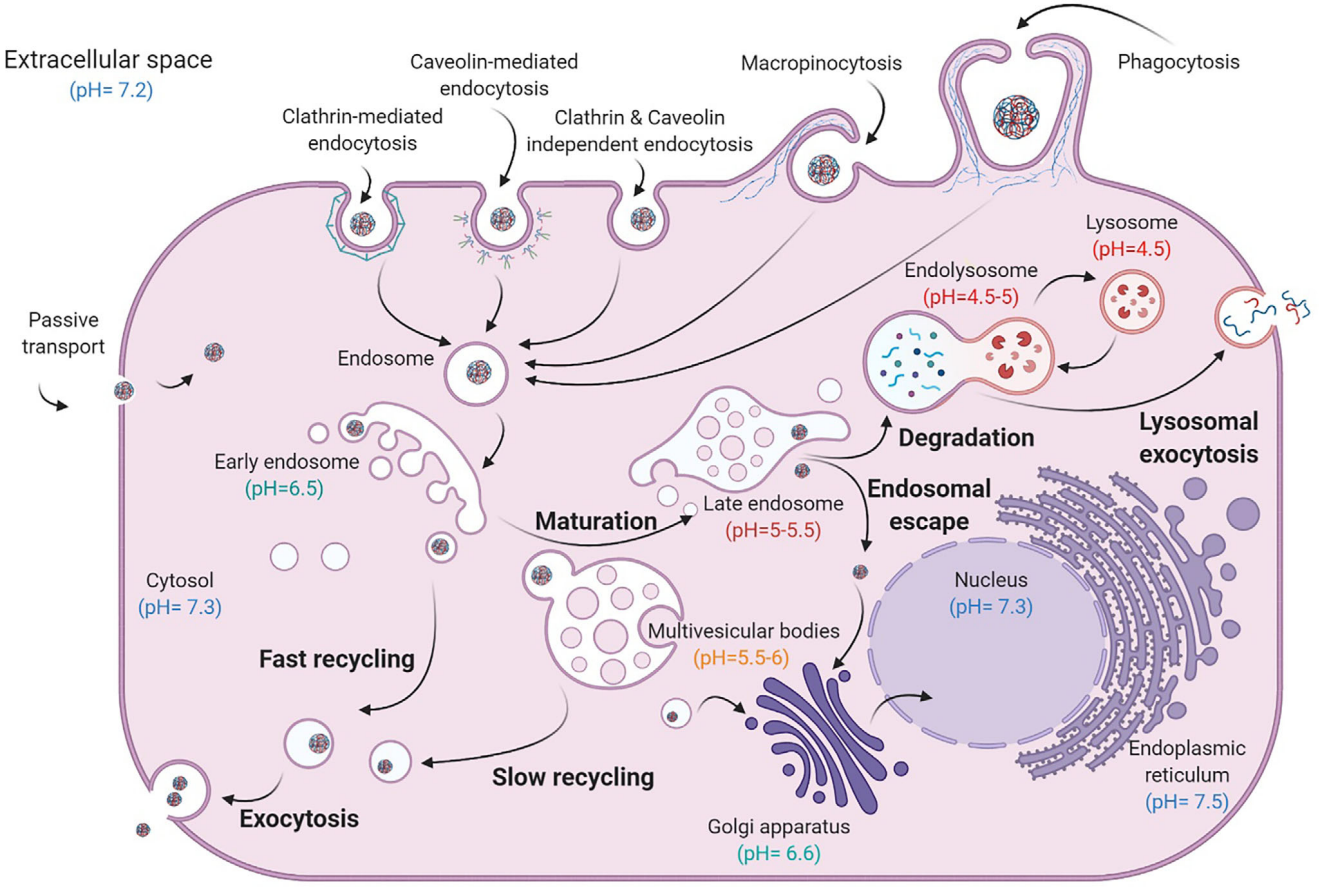
Uptake pathways and subsequent intracellular trafficking mechanisms. pH is indicated at different stages within corresponding cellular compartment (Created with BioRender.com).

The next challenge is cellular uptake. As previously mentioned, NAs are unable to passively pass through a negatively charged cell membrane. Thus, complexation with a carrier system is a prerequisite for condensing NA payloads and facilitating cellular uptake. Different uptake routes include clathrin‐mediated endocytosis, caveolae‐dependent endocytosis, phagocytosis, and micropinocytosis.^[^ ^11^, ^12^ ^]^ Since multiple pathways are often simultaneously involved,^[^ ^13^ ^]^ understanding the uptake mechanism is crucial for optimizing intracellular trafficking, biodistribution, and overall carrier performance (**Figure** **2**).^[^ ^14^, ^15^ ^]^

Following uptake polyplexes are typically trafficked to *early endosomes*, which undergo maturation into *late endosomes* by progressive acidification. Without timely interruption, they eventually fuse to lysosomes to form *endolysosomes*, where their contents are degraded by lysosomal enzymes.^[^ ^16^ ^]^ Thus, the next obstacle in intracellular trafficking of polyplexes is *endosomal escape* of NAs into the cytosol before degradation occurs. The exact mechanism of endosomal escape is still under debate. An early hypothesis, known as the *proton sponge effect*, proposed that the buffering capacity of cationic carriers leads to increased proton influx, causing osmotic swelling and rupture of the endosomal membrane.^[^ ^17^ ^]^ However, more recent studies challenge this theory, instead suggesting that cationic polymers disrupt endosomal membranes directly through pH‐dependent membrane permeabilization, a process that intensifies as endosomal acidity increases.^[^ ^18^ ^]^

For ribonucleic acid payloads, such as siRNA and mRNA, reaching the cytosol is sufficient to perform their biological function. In contrast, plasmid DNA (pDNA) must overcome an additional barrier of the nuclear membrane where it can either be directly transcribed, or in the case of genome editing, integrated into the target location in the genome.^[^ ^19^ ^]^ In both scenarios, the final critical step is the release of the NA from its carrier, ensuring its availability at the intended intracellular site of action.

The fate of polymeric carriers upon release of the NA payload inside cells represents the next challenge. Depending on whether the carrier is degradable or nondegradable, different mechanisms are involved in its elimination, and degradation products may interact differently with the biological environment.^[^ ^20^, ^21^ ^]^ Therefore, toxicity arising not only from intact polyplexes but also from their degradation products, which should be considered in designing polymeric carrier systems.^[^ ^22^, ^23^, ^24^ ^]^ Moreover, immunogenicity is particularly critical when immune cells are targeted, as toll‐like receptors can recognize polyplexes as a threat.^[^ ^25^, ^26^, ^27^ ^]^ Notably, immune responses can be triggered either by the NA payload or by specific structural features of the polymeric carrier.^[^ ^28^, ^29^ ^]^ While the mechanisms of immune activation triggered by NAs are well‐defined, responses induced by polymeric carriers remain insufficiently understood. A recent review has highlighted how specific structural features of polymeric carriers such as charge, hydrophobicity, and molecular weight can affect immune recognition.^[^ ^30^ ^]^ Nevertheless, more systematic side by side studies are needed to directly correlate polymer physicochemical properties with immune recognition and to elucidate the underlying mechanisms. Overall, efficient NA delivery requires the careful balance between transfection performance, carrier degradability, safety, and immune compatibility tailored to both the payload and the target cell.

#### 1.1.2 Physiological and Biological Barriers to In Vivo Nucleic Acid Delivery

In vivo NA delivery via intravenous administration faces multiple barriers, including clearance by the liver, kidney, and spleen, destabilization in the glomerular membrane, opsonization and degradation by phagocytes, aggregation with serum proteins, and physical obstacles such as the vascular endothelial barrier.^[^ ^31^, ^32^, ^33^ ^]^ Opsonization and subsequent phagolysosomal degradation by innate immune cells can cause rapid elimination of polyplexes.^[^ ^34^, ^35^ ^]^ Physical barriers, such as endothelial tight junctions and the extracellular matrix (“glycocalyx”), further hinder tissue penetration.^[^ ^36^ ^]^

In addition to delivery efficiency, safety of polyplex delivery is crucial. Accumulation of intact or nondegradable polymers can lead to cytotoxicity and release of reactive oxygen species (ROS) that may harm surrounding tissue. Immunogenicity poses another challenge, as mentioned previously.^[^ ^37^, ^38^ ^]^ While immune interactions often impair transfection efficiency, they can be harnessed therapeutically, for example to stimulate T‐cell recruitment in immunosuppressed tumor microenvironments.^[^ ^39^ ^]^ Thus, rational carrier design must balance stability, tissue selectivity, and immune compatibility to achieve safe and effective in vivo delivery.

## 2 Applying Structural Organization Principles to Payload‐Specific Carrier Design: Structure–Function Relationships

**Figure 3:**
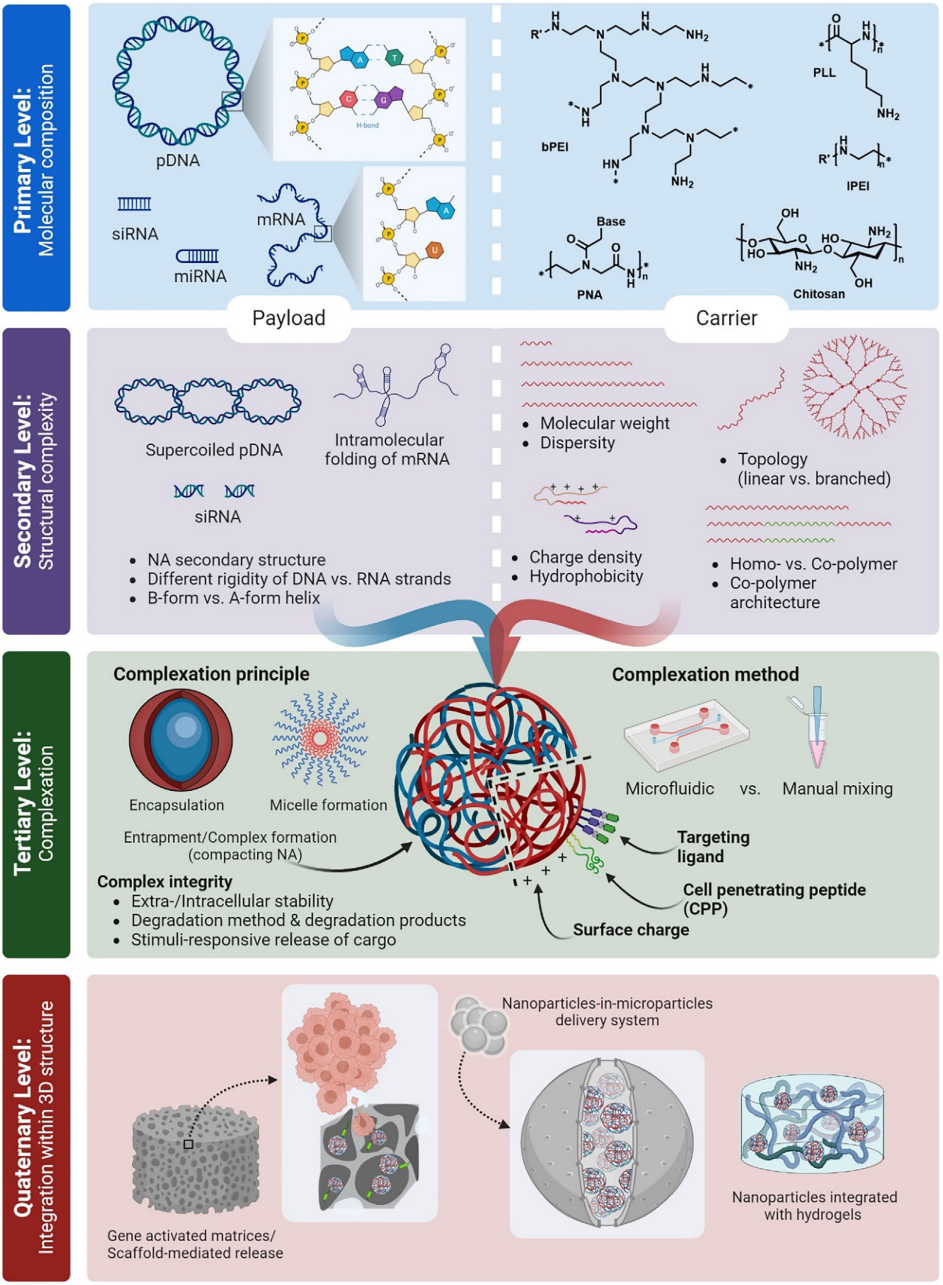
Four levels of structural complexity applied in polymeric carrier systems for NA delivery, to be considered for rational design with respect to specific functions. The primary level indicates the molecular composition of various NA types as payload and chemical structure of a few prominently used polymers as carrier. Structural complexity is reflected in the secondary level in each case. The tertiary level covers complexation method and complex forms of the carrier and payload illustrated in red and dark blue color, respectively. In the quaternary level, the polyplexes are further loaded within a 3D structure, also known as gene activated matrix for a prolonged sustained release. pDNA: plasmid DNA, siRNA: short interfering RNA, miRNA: microRNA, mRNA: messenger RNA, lPEI: linear Polyethylenimine, bPEI: branched PEI, PNA: Poly NA, PLL: poly‐L‐lysine, NA: nucleic acid (Created with BioRender.com).

A rationally designed carrier system must accommodate a given payload to overcome or bypass its corresponding biological barriers as briefly described above. Here, the key question is how a particular function could be fulfilled regarding structural properties of polyplexes. Thus, the features of both components must be considered when designing a NA delivery system. However, the majority of studies in this field have only focused on different carrier properties, regardless of the type of payload, resulting in the use of identical formulations, for delivery of chemically distinct entities with drastically different structural properties.^[^ ^40^ ^]^ In other examples, a carrier which was previously established for pDNA polyplex formation^[^ ^41^ ^]^ was tested for delivery of a different type of NA, i.e., siRNA, with a distinct chemical nature and different size.^[^ ^42^ ^]^ Deep understanding of the structural/mechanistic differences between various payloads is expected to facilitate development of the next generation of carrier systems, specifically designed for a certain payload type.^[^ ^8^ ^]^ In this chapter, the structural properties of carrier, NA payload, and resulting polyplexes are elaborated through different levels of organization, in analogy to the four levels of protein structure (**Figure** **3**). This representation is meant to help logically categorize properties, starting from basic chemical structure of each entity, followed by a step‐by‐step strategy to accommodate the increase of complexity of carrier systems in larger scales (Figure 3). It should be noted that we do not aim to provide an exhaustive overview over all relevant literature but rather guide decisions in NA delivery system design by selected examples.

Moreover, in this review, we define polymeric carriers as synthetic polymers composed of repeating chemical units (e.g., polyethylenimine, poly(β‐amino ester), and poly(amidoamine) dendrimers) that complex with NAs through electrostatic or covalent interactions. By contrast, self‐assembled NA nanostructures such as DNA origami are classified here as NA‐based nanomaterials and are discussed separately in Section 3.4.

### 2.1 Primary Level: Molecular Composition

#### 2.1.1 Payload Molecular Composition

In NA delivery, a payload is by definition a biomolecule which transfers genetic information to target living cells in form of DNA or RNA. These biopolymers are generally composed of different nucleotides as monomers, covalently connected in the form of linear macromolecules. The so‐called “backbone” of NAs is composed of alternating sugar/phosphate units, with the phosphate groups as the main determinant of their strong negative charge, a key property for carrier design in gene transfer. The nucleotide identity corresponds to the nucleobase linked to the nucleotide's sugar moiety. Their sequence of nucleobases in 5′ to 3′ direction determines the genetic information encoded by the NA molecule. In general, ribonucleic acids have one extra hydroxyl group on the 5‐membered sugar part of their repeating units compared with deoxyribonucleic acids (Figure 3). The additional hydroxyl group at 2′ position of ribose results in increased stability and thus melting temperature of compact A‐form helix in RNA duplexes compared to B‐form helix in double strand DNA structure.^[^ ^43^ ^]^ In the following paragraphs we elaborate the molecular identity of the major NAs used in therapeutic applications and emphasize their distinct structural features (Table S1, Supporting Information)

***i) Plasmid DNA*** (pDNA) is a circular double‐stranded DNA molecule, originally derived from bacteria, where it functions as an autonomous replicating unit. Some of the typical features of this closed‐ring biomolecule are an origin of replication (bacterial; in some exceptions also eukaryotic), antibiotic resistance markers facilitating the isolation of plasmid‐harboring cells, and a promoter as a transcription initiator upstream of the target sequence to be transcribed, mostly the so‐called gene of interests, or GOI. Such recombinant plasmids have been the workhorse of genetic engineering for many decades. When introducing a pDNA to a living eukaryotic cell, it must first enter the nucleus, in order to be transcribed by host RNA polymerases. Subsequently, newly formed mRNA molecules will be transported from the nucleus to the cytoplasm, where they are translated into proteins by ribosomes, completing expression of the transferred gene.^[^ ^44^ ^]^ Such pDNAs are therefore often referred to as expression vectors. Of note, in this context the definition of expression “vector” is a biomolecule which holds the sequence of the gene of interest, which is not identical to the “chemical vector” for carrying the biomolecule itself.

***ii) Messenger RNA*** (mRNA) is another molecular entity suited to transfer genetic information to cells. Although mRNA is known to be single stranded, relatively often they also form inter‐/intramolecular base paring, causing formation of partial double strand or secondary structures. Traditionally mRNA was known to be less stable and highly sensitive to fast degradation, which restricted its applications in initial gene therapy studies. However, advances in mRNA synthesis by in vitro transcription (IVT) technology enabled incorporation of structural modifications to transcripts, which were beneficial in addressing many of the aforementioned limitations. Implementing the phage RNA polymerases, such as T7 or SP6, various types of canonical or chemically modified ribonucleotides can be implemented as monomeric substrates in enzymatic bio‐polymerization of mRNA molecules. Noteworthy, mRNA is recently favored in many biotechnology studies, due to its safety, i.e., no potential risk of genotoxicity, meanwhile increased stability and safe degradation, high translatability, rapid and transient expression immediately after delivery, as well as rapid and scalable production.^[^ ^45^ ^]^ Hence, this biomolecule has gained prominence as “a new class of drugs”.^[^ ^46^ ^]^ The most prominent example is the recent mRNA based‐SARS‐Cov‐2 vaccine, that was rapidly developed and broadly administrated world‐wide.

Some of the structural elements of an IVT‐synthesized mRNA molecules, which accommodate the above‐mentioned features, are 5′‐cap, 3′‐polyA tail, 5′and 3′‐untranslated regions (UTRs), various chemically modified nucleotides. All of these features, directly or indirectly contribute to increase stability, translatability and to eliminate unwanted immune response to exogenous mRNA, in part by mimicking features of naturally occurring mRNA.^[^ ^46^ ^]^ The 5′ end modification, for instance, includes addition of a GpppG structure either co‐ or post‐transcriptionally to the newly transcribed mRNA molecules. The so called cap structure protects mRNA molecules from RNase degradation, support the cap‐dependent ribosomal translation, and reduces the immunogenicity stimulated by 5′phosphate‐recognizing intracellular receptors, e.g. RIG‐I.^[^ ^28^ ^]^ Chemical modification of nucleotides influences the structural properties of mRNA molecules by promoting or destabilizing formation of hydrogen bonds with other nucleotides. Noncanonical base pairing can result in the formation of partial double strand structures and thus can alter the mRNA molecules secondary structure.^[^ ^47^, ^48^, ^49^ ^]^ Whether the chemically modified mRNAs with their distinct properties impact complexation process with a carrier and the way it impacts the physicochemical properties of the final polyplex product remains to be explored. In contrast to pDNA, the intracellular target location for mRNA transfection is the cytoplasm, where it can be immediately translated.^[^ ^50^ ^]^

***iii) Self‐amplifying RNAs*** (saRNA) are derived from positive‐sense alphavirus genomes. Compared to conventional mRNA, transfection with saRNA results in prolonged overexpression over time, i.e., weeks to months. The saRNA vaccine platforms were proven to be effective in protective immunization against infectious diseases including influenza, Rabies, Ebola, HIV and recently SARS‐Cov‐2 based on preclinical studies.^[^ ^51^ ^]^ The intracellular RNA‐dependent RNA replication is achieved by four nonstructural proteins (nsP1‐4), encoded in *cis* 3′ to the gene of interest and a selection marker. Therefore, the delivery of very low doses of saRNA to target cells can effectively generate a high dose of the desired therapeutic protein, which assists in meeting the therapeutic window defined in clinical settings.^[^ ^52^ ^]^ While sharing the same chemistry with mRNA, the saRNA is enriched in secondary structures, particularly in conserved sequence element (CSE) promoter regions, where an RNA‐dependent RNA polymerize recognizes and initiates the transcription process. Another remarkable structural feature of saRNAs is their large molecular size, adding up the gene of interest sequence to 7.3 kb of the vector coding for the replicase, which make the overall size of the resulting RNA much larger than an average mRNA molecule.^[^ ^52^ ^]^

***iv) Small RNAs***, including **microRNAs (miRNAs), small interfering RNAs (siRNAs), short hairpin RNAs (shRNAs), and ribozymes** are intracellularly processed from longer precursor RNAs transcribed from the genome and play a central role in gene regulation through RNA silencing (or RNA interference). This post‐transcriptional regulation can occur via cleavage of mRNA transcripts or inhibition of translation, both mediated by base pairing between the small RNA and its target. Among them, miRNAs and siRNAs are the most prominent examples, recognizing and binding to mRNA molecules to induce degradation and thereby achieve transient gene silencing.^[^ ^53^ ^]^ Exogenous introduction of their large precursors, either as pDNA or in vitro transcribed RNA (IVT‐RNA), follows the delivery principles outlined above. However, short chemically synthesized RNAs are routinely transfected in cells in the form of preannealed double‐stranded siRNAs or miRNA mimics, with applications ranging from loss‐of‐function analysis of individual targeted genes, to large‐scale screens using short RNA libraries.^[^ ^54^ ^]^ These short RNAs have a transient effect on gene activity, typically lasting several days. Their stability is often enhanced by use of chemically modified nucleotides. Their comparatively small size offer the opportunity to tailor carrier systems especially accommodating this type of NA, and functionally delivering it to either the cytoplasm and/or nucleus of cells.^[^ ^55^ ^]^

Ribozymes are catalytically active RNA molecules that can cleave or splice target RNA at specific nucleotide sequences, thereby regulating gene expression at the post‐transcriptional level.^[^ ^56^ ^]^ Ribozymes are relatively small, typically 30–150 nucleotides in length (≈10–50 kDa), which allows them to fold into compact secondary structures required for catalysis.^[^ ^56^ ^]^ The potential therapeutic applications of ribozymes in treating muscular dystrophy were reported in a recent study.^[^ ^57^ ^]^ However, their susceptibility to nucleases and limited intracellular delivery remain major bottlenecks for clinical translation. Therefore, advanced carrier systems are essential to improve ribozyme stability and biological function.

***v) Single‐guide RNAs (sgRNAs)*** are central to CRISPR‐based genome editing and require efficient intracellular delivery to direct the nuclease to the intended genomic locus. In practice, sgRNAs are often co‐delivered with a Cas nuclease, which can be introduced in different formats: as plasmid DNA encoding the nuclease, as mRNA for cytosolic translation, or as a preassembled ribonucleoprotein (RNP) complex. Each approach poses distinct delivery challenges—plasmid DNA must reach the nucleus, mRNA must remain stable in the cytoplasm for translation, and RNPs require direct cytosolic access but provide the fastest and most transient activity.^[^ ^3^ ^]^ These diverse formats illustrate the importance of payload‐specific design of polymeric carriers, which must be tailored to support the stability, localization, and release requirements of each CRISPR component.

***vi) Other oligonucleotides*,** *including* ***single‐stranded DNA (ssDNA), antisense oligonucleotides (ASOs), and aptamers*,** present delivery requirements distinct from those of plasmid DNA, siRNA, and mRNA. ssDNA are utilized as ASOs to regulate gene expression or as donor templates in genome editing applications, where stability against nucleases and efficient nuclear localization are critical. Aptamers, typically short ssDNA or ssRNA molecules, fold into defined 3D structures that enable high‐affinity binding to target proteins.^[^ ^58^, ^59^ ^]^ While aptamers are most often employed as targeting ligands on carrier surfaces, they can also function directly as therapeutic cargos, as demonstrated by the FDA‐approved RNA aptamer Pegaptanib.^[^ ^60^ ^]^ Several ASO drugs, such as Nusinersen (for spinal muscular atrophy)^[^ ^61^ ^]^ and Eteplirsen (for Duchenne muscular dystrophy),^[^ ^62^ ^]^ are already clinically approved, underscoring their therapeutic relevance and highlighting the need for payload‐specific adaptation of polymeric carrier systems.

It should be noted that certain NA‐based probes, such as DNA molecular beacons or FRET probes, are specifically designed for imaging or diagnostic applications.^[^ ^63^, ^64^ ^]^ While these systems share some delivery challenges with therapeutic NAs, their use is primarily restricted to intracellular detection rather than treatment, and therefore they are not covered in detail in this review.

#### 2.1.2 Types of Polymers Used as Nucleic Acid Carriers

**Figure 4:**
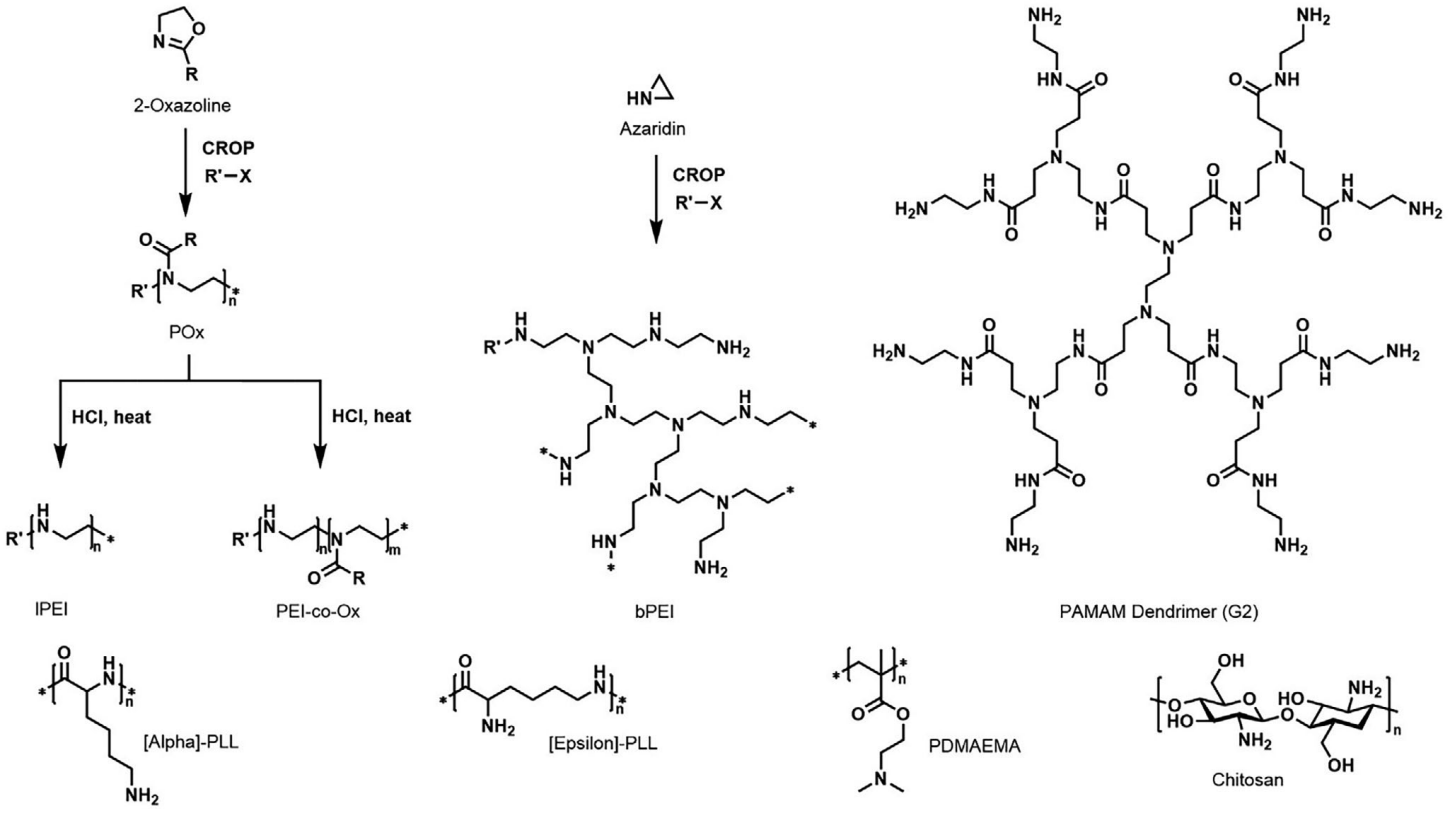
Schematic representation of the synthesis and structure of various polymer classed used in the context of gene transfection.

For the overall performance of an NA delivery system, the choice of cationic carrier material is of paramount importance. Here, a few examples of commonly used compounds are highlighted. Early work on diethylaminoethyl‐dextran and its enhancement of transfection^[^ ^65^ ^]^ paved the way for nonviral NA delivery using cationic polymers (Table S2, Supporting Information). Following this discovery, a considerable number of studies focused on polyamino acids. Here, polymers like poly(L‐arginine) or poly(L‐ornithine) were used, but most prominently poly(L‐lysine) (PLL) was investigated for its use in NA delivery.^[^ ^66^ ^]^ As the polymer consists of naturally occurring amino acids, it leads to a low or negligible immune response.^[^ ^67^ ^]^ Independent whether α‐PLL or ε‐PLL (**Figure** **4**) is used, the polymer possesses a high density of primary amino groups that can efficiently condense NAs.^[^ ^68^ ^]^ Another type of poly(cation) that was established for transfection in the 1990s were poly(amidoamine) (PAMAM) dendrimers.^[^ ^69^ ^]^ In contrast to linear polymers with a certain molar mass distribution, these dendrimers have a perfectly defined structure featuring a highly branched, globular morphology. PAMAM features a mixture of tertiary amino groups (within the dendrimer) and primary amino groups (on the surface) and its size is controllable by the number of generations added during synthesis. However, compared to conventional polymer synthesis, PAMAM dendrimer production is rather complicated and demanding independent of whether a divergent,^[^ ^70^ ^]^ or a convergent^[^ ^71^ ^]^ strategy is used. The highly functional surface can be used to attach different ligands or PEG chains to modulate NA complexation and cellular uptake, among others.^[^ ^72^ ^]^ PAMAM dendrimers were successfully used in in vivo studies in the last decade.^[^ ^73^ ^]^ In a recent study, DNA and RNA nanocubes were complexed with different generations of amine‐terminated PAMAM dendrimers to evaluate delivery efficiency and immunogenicity. To gain deeper insight into polyplex structure‐function relationships, molecular dynamics (MD) simulations were performed, and side by side comparisons showed that dendrimer branch size critically influenced delivery efficacy and immune recognition.^[^ ^74^ ^]^ Unlike earlier work on PAMAM‐mediated siRNA and pDNA delivery,^[^ ^75^ ^]^ this study reported no significant differences in cytotoxicity among dendrimer generations.^[^ ^74^ ^]^

One of the most successful and well‐studied polymers in the context of NA delivery is poly(ethylene imine) (PEI), which was first used for transfection in 1995.^[^ ^76^ ^]^ PEI is often referred to as the “gold standard” in nonviral transfection, and is frequently used as a benchmark for newly synthesized materials, enabling efficient gene expression in HEK293T and CHO cells.^[^ ^77^, ^78^ ^]^ PEI can be subdivided into two classes: branched PEI (bPEI), and linear PEI (lPEI),^[^ ^79^ ^]^ with distinct differences in structure and properties between the two. bPEI is produced by a cationic ring opening polymerization (CROP) of aziridine, a highly uncontrolled process leading to branching and a mixture of primary, secondary and tertiary amino groups.^[^ ^80^ ^]^ While substituted azaridines can be used to produce linear polymers, a more commonly employed way to produce lPEI is by hydrolysis of poly(2‐oxazoline)s (POx).^[^ ^81^ ^]^ The linear structure and (usually narrow) molar mass distribution of the precursor is maintained throughout hydrolysis and the final polymer possesses only secondary amino groups. An additional advantage is that the properties of the polymer can be modulated by incomplete hydrolysis leading to POx‐EI‐copolymers.^[^ ^82^ ^]^ Such copolymers possess a lowered charge density and an associated decrease in cytotoxicity, which however also leads to a limited transfection ability. To further modify the properties of such polymers, various functionalization strategies are described in the literature.^[^ ^79^ ^]^

Due to its high charge density and beneficial p*K*_a_ value(s) leading to limited protonation at physiological pH, PEI‐based polyplexes show relatively efficient endosomal escape.^[^ ^18^ ^]^ This was initially reported to be a result of the so called “proton‐sponge‐effect”, describing a burst release of endosomal compartments as a result of increased osmotic pressure.^[^ ^17^ ^]^ However, more recent investigations favor polymer‐induced membrane interaction as mechanistical basis for endosomal escape,^[^ ^18^ ^]^ leading to hole formation,^[^ ^83^ ^]^ or permeabilization of the endosomal membrane.^[^ ^84^ ^]^ The polyplexes formed by branched or linear PEI were shown to have distinct physicochemical properties which resulted in different transfection efficiencies. bPEI forms smaller polyplexes, which can be favorable for circulation in in vivo applications.^[^ ^85^ ^]^ However, lPEI‐based polyplexes were found to be lower in toxicity in vivo.^[^ ^17^ ^]^ In general, lPEI‐based polyplexes show increased transfection both in vitro and in vivo,^[^ ^86^ ^]^ but this also heavily scales with preparation conditions of the polyplex (e.g., presence of salt).^[^ ^77^, ^78^, ^87^ ^]^ The secondary amine groups of PEI can also be functionalized to further alter its properties. Oxidation can, for instance, be used to induce degradability and increase biocompatibility.^[^ ^88^ ^]^ In a recent study, PEI structure was modified using a multicomponent split‐Ugi reaction method, which was employed to synthesize a library of 155 polymers formulated into polyplexes to evaluate a structure‐function relationship based on endosomal escape and transfection efficiency. Polyplexes were developed to deliver mRNA with different sizes to lung endothelium and immune cells such T cells. Screening this library, they identified the best formula with the least toxicity and improved mRNA delivery to target immune cells in lung.^[^ ^89^ ^]^ Most recently, **branched PEI** has been employed to generate compact self‐amplifying RNA (saRNA) vaccines, where single‐molecule PEI/saRNA polyplexes of ≈30 nm achieved enhanced activity in vitro and in vivo and elicited robust immune responses at lower doses in vaccination models.^[^ ^90^ ^]^

A further relevant material, which is based on acrylic monomers is poly(dimethyl aminoethyl methacrylate) (PDMAEMA) and was first used in transfection applications in 1996.^[^ ^91^ ^]^ The polymer features only tertiary amines and its transfection efficiency is dependent on it molar mass.^[^ ^92^ ^]^ In addition to the inherent pH‐responsibility of polycations, PDMAEMA is also a temperature sensitive material.^[^ ^93^ ^]^ One noteworthy advantage of PDMAEMA as a building block for NA delivery vectors is its easy incorporation into complex macromolecules like block copolymers.^[^ ^77^ ^]^ Harsh hydrolysis conditions, as for instance necessary in the production of lPEI do not restrict material design, and DMAEMA can easily be copolymerized with other acrylic monomers for instance using reversible deactivation radical polymerization (RDRP) techniques, enabling synthesis of block copolymers,^[^ ^94^, ^95^ ^]^ graft architectures,^[^ ^96^ ^]^ star copolymers,^[^ ^97^ ^]^ or the introduction of defined end groups for further coupling reactions, e.g., with PEG,^[^ ^98^ ^]^ which can be harnessed for NA delivery. For instance, a study developed nanohybrids by conjugating methoxy‐poly(ethylene glycol) (mPEG) to graphene oxide and combining them with PDMAEMA, which enabled efficient siRNA delivery with low cytotoxicity and offered additional optical properties, making them attractive for combined photothermal and NA therapy applications.^[^ ^99^ ^]^

Most of the materials presented so far are nonbiodegradable. However, degradability upon endosomal release and unpacking of NA in the cell cytoplasm is a highly favorable property, as it avoids potential unintended changes in cellular behavior.^[^ ^100^ ^]^ Poly(β‐amino esters) (PBAE)s are a promising class of degradable biomaterials that were first utilized for NA delivery in 2000 by the group of Langer.^[^ ^101^ ^]^ As their name suggests, they contain both, amino and ester functions along their polymeric backbone, which renders them cationic and degradable.^[^ ^102^ ^]^ While there are multiple routes leading to PBAE, the most common way is an aza‐michael addition between primary amines and bis‐functional acrylic compounds.^[^ ^103^ ^]^ A plethora of commercially available building blocks enables straightforward variation of polymer composition and properties to suit specific needs.^[^ ^104^ ^]^ In particular, PBAEs have demonstrated strong potential for mRNA delivery, enabling efficient protein expression. In a recent study, a library of 55 PBAE polymers was synthesized and screened for mRNA delivery efficiency, with optimized N/P ratios identified through in vivo testing. The resulting polyplexes demonstrated sustained mRNA expression for up to two weeks, while restricting expression to the injection site and showing no liver toxicity.^[^ ^105^ ^]^ Among the tested library, polymers incorporating amine 8 (5‐amino‐1‐pentanol) achieved the highest mRNA delivery. This was attributed to the amphiphilic nature of the monomer, where the hydroxyl group and flexible alkyl chain enhanced interactions with cell membranes, thereby promoting cellular uptake and prolonged expression. In vivo testing of the selected polyplexes revealed varying levels of immune response, depending on the monomer composition and resulting physicochemical properties of the polymers.^[^ ^105^ ^]^

Lastly, self‐assembled DNA nanostructures, commonly referred to as DNA origami, can also serve as carriers for NA delivery such as siRNA by protecting them from degradation and facilitating cellular uptake.^[^ ^106^ ^]^ While we briefly discuss DNA origami in Section 3.4, in this review, NAs are primarily considered as payloads rather than carriers, with the main focus placed on synthetic cationic polymers for NA delivery.

### 2.2 Secondary Level: Structural Complexity

#### 2.2.1 Physicochemical Characteristics of Payload

There are remarkable differences in structure of the four categories of NA payloads, i.e., pDNA, mRNA, extremely large self‐amplifying RNA (saRNA), and small RNAs. Aside from their different chemistry, these biomolecules also have remarkably different physicochemical properties. Examples are size, secondary structure, topology, molecular rigidity, and charge density. In the following we briefly explain these features.

pDNAs are typically quite large molecules of 3000–10 000 base pairs (bp) (≈ 650 g mol^−1^ per base pair) but larger representatives like bacmids^[^ ^107^ ^]^ possess more than 100 000 bp, thus exceed 10^6^ g mol^−1^ in molecular weight. Synthetic mRNA molecules with 500–10 000 nucleotides (nt) length (≈320 g mol^−1^ per nucleotide) are comparatively small. Of the same nature, saRNA molecules are composed of more than 11 000 nt, which is larger than an average mRNA molecule. In contrast, siRNA and miRNA are almost 200 times smaller than a typical pDNA used for NA delivery, since they only contain 20–30 bp (≈13 to 20 × 10^3^ g mol^−1^).

To estimate the hydrodynamic diameter of different NAs, i.e., their dimension in aqueous solution, we need to also consider their intrinsic secondary structure and topology. pDNA and siRNA are known to have double‐strand structure, whereas mRNA is typically considered to be single stranded. Nonetheless, depending on the nucleotide sequence, intramolecular interactions in mRNA can lead to formation of partial double‐stranded conformations, and eventually2D or 3D structures. This is particularly pronounced for saRNA molecules, which are rich in secondary structures, essential for their self‐replication mechanism.^[^ ^108^ ^]^

Notably, physical properties of double strand structure vary in RNA compared to DNA. Studies have shown that a double‐stranded RNA (dsRNA) molecule is stiffer than dsDNA of the same size and sequence,^[^ ^109^ ^]^ potentially influencing the choice of carrier. Moreover, dsRNA was found to be more rigid than single‐stranded RNA (ssRNA) of the same size with the same nucleotide sequence. The differences in payload chain rigidity affected the mechanism of complex formation, and multimolecular assembly formation, which was only investigated for ssRNA with more flexible chains.^[^ ^110^ ^]^ Of note, pDNA can also form 3D structure, by formation of a particular topography called “supercoil”, which dramatically reduces its molecular dimension compared with “relaxed” conformation. Therefore, the average approximate dimensions for pDNA, mRNA and siRNA would be hundreds of nm, tens to hundreds of nm, and < 10 nm, respectively.^[^ ^111^ ^]^ More details on the effect of DNA, and RNA self‐assembled secondary structures on biophysical properties of the corresponding polyplexes, with particular focus on their effects on immune response modulation are discussed elsewhere.^[^ ^112^ ^]^

Since overall negative charge of NA molecules depends on the number of phosphate groups, i.e., directly corresponding to the number of nucleotides, the size of the anionic polynucleotide chain will affect the strength of electrostatic interaction or binding affinity with the cationic polymer, and is an obvious and decisive physical property of the payload relevant to the choice of the carrier system.

#### 2.2.2 Physicochemical Characteristics of the Carrier

A polymeric carrier relies on its physicochemical properties to meet functional requirements for NA delivery, and subtle structural changes can have dramatic impacts on their biological performance (Table S3, Supporting Information).^[^ ^113^ ^]^ The following paragraphs outline how payload parameters such as charge density, molecular weight, rigidity, and topology influences key NA delivery functions.

***i) Cationic charge*** of polymers is essential for NA condensation, protection against nucleases and serum stability.^[^ ^114^ ^]^ Electrostatic interactions with cationic polymers neutralize intramolecular repulsion of anionic NAs, analogous to DNA condensation by histones in chromatin.^[^ ^115^, ^116^ ^]^ The degree of ionization, defined by the polymer's p*K*_a_ and charge density, is critical for balancing stability, uptake, and release, since excessive charge can cause cytotoxicity or hinder unpacking.^[^ ^5^, ^117^, ^118^ ^]^ As pH varies during intracellular trafficking (neutral in early endosomes to <4.5 in endolysosomes as indicated in Figure 2), carriers are often designed to respond to these changes and release payloads under acidic conditions.^[^ ^119^, ^120^ ^]^ For example, a recent study screening a library of 168 PBAE carriers showed that delivery efficiency and toxicity were strongly dependent on backbone amine chemistry rather than end‐capping groups. In particular, polymers with a p*K*_a_ of 6.2–6.5 showed high siRNA delivery efficiency with low cytotoxicity, whereas other series displayed comparable efficacy but elevated toxicity. Importantly, differences in delivery were not explained by uptake but likely by intracellular trafficking or stability, underscoring how subtle tuning of polymer charge and p*K*_a_ critically modulate performance.^[^ ^121^ ^]^

***ii) Molar mass*** of the polymer can also massively influence the success of gene transfer.^[^ ^122^, ^123^ ^]^ These studies have suggested that molar mass is positively correlated with transfection efficiency. On the downside, high molar mass often increases cytotoxicity, as a result of higher affinity to cellular membranes. In a systematic study, Blakney et al. screened a library of polymers with variable molar masses as well as charge density (in terms of ethylene imine content) of partially hydrolyzed poly(2‐ethyl‐2‐oxazoline)/poly(ethylene imine) copolymers, to find the optimal formula for different types of NAs. They found that polymers with high molar masses, 83000 g mol^−1^ and 72000 g mol^−1^, resulted in the highest efficiency for delivery of pDNA and large RNA molecules (i.e., self‐amplifying replicon RNA), respectively, whereas in the case of mRNA with a smaller size compared to the above mentioned NAs, polymers with medium molar mass, i.e., 45 000 g mol^−1^, lead to highest transfection efficiency.^[^ ^122^ ^]^

In another study, a library of PDMAEMA, and DEAEMA copolymers with different properties including molar mass and sizes of alkyl side chains was screened using a fully automated high throughput workflow, in order to find the most effective formula for mRNA delivery in vitro. Cell lines including Huh7, HeLa, H359, and HepG2 were selected for biological evaluation. Encapsulation of mRNA molecules, NPs size, cell viability, and transfection efficiency were measured as key readouts. Findings of this study revealed that copolymers with the lowest molecular weight led to the highest transfection efficiency and cell viability, also when compared to conventional PEI transfection. On the other hand, the highest encapsulation efficiency was observed for copolymers with shorter alkyl side chains and higher cationic density.^[^ ^124^ ^]^ Larger molar mass of the carrier‐forming polymer was reported to directly correlate with particles size, DNA binding affinity, and the number of DNA molecules per particle.^[^ ^125^ ^]^

***iii) Hydrophobicity*** of polymer backbones or, alternatively, addition of extra hydrophobic side chains were reported to lead to enhanced transfection efficacy, while on the downside triggering cytotoxic effects.^[^ ^126^, ^127^, ^128^ ^]^ An essential part of gene transfection is successful endosomal escape, i.e., passing through the endosomal membrane, a process that can be enhanced by hydrophobic subunits. Hydrophobic chains of polymers can however also interact with the outer layer of the cellular membrane causing disruption and cell death.^[^ ^129^, ^130^ ^]^ Red blood cells are usually particularly affected by this type of toxicity, and hence should specifically be considered in gene therapy applications.^[^ ^131^ ^]^

The size of alkyl side chains was recently reported to play a crucial role in the formation of stable mRNA‐encapsulated polymer‐lipid nanoparticles. A library of ionizable amino‐polyesters with varying alkyl side chain length and degrees of unsaturation were synthesized via ring‐opening polymerization and tested for mRNA delivery. The results of in vitro and in vivo evaluation revealed that alkyl side chain length is a crucial parameter in stability of mRNA NPs and efficient delivery. Notably, tissue‐specific delivery was reported depending on side chain length for example, nanoparticles with 4–5 carbon side chains preferentially targeted the spleen and lungs, while those with 7–9 carbon chains showed enhanced liver targeting.^[^ ^132^ ^]^ Together, such studies highlight the importance of molecular weight and composition of polymers not only in efficient NA delivery, but also in potential ligand‐free tissue‐specific targeting of nanoparticles.

***iv) Topology*** of polymers refers to their linear, branched, hyper‐branched, or grafted architecture. Provided that the polymer is positively charged, this architecture would remarkably affect the density and distribution of charge, and subsequently influence functions such as NA condensation and condensation strength, polyplex stability, transport through the membrane as well as endosomal swelling and endosomal escape.^[^ ^133^, ^134^ ^]^ For example, it has been reported that highly branched PEI NPs are more effective in terms of endosomal escape by inducing higher osmotic swelling than those with a linear structure.^[^ ^135^ ^]^ In contras, another study by Wagner and colleagues demonstrated remarkably greater transfection efficiency of linear PEI complexed with DNA compared to branched PEI tested in vitro.^[^ ^87^ ^]^ An intermediate version was reported, containing linear PEI (and hence only secondary amines) in a hyperbranched topology, effectively decreasing undesired toxicity while maintaining transfection efficiency.^[^ ^136^ ^]^ Also grafted topologies, i.e., bottle brush copolymers were described to boost gene transfection, by both increased transfection efficiency as well as lowered cytotoxicity.^[^ ^96^, ^137^ ^]^

In case of using linear block copolymers, the order of different blocks with respect to each other affects the final outcome of NA delivery, as investigated systematically using a library of polymers by Breunig and colleagues.^[^ ^138^ ^]^ The type of functional moieties incorporated within each block as well as the ratio of the two blocks with respect to each other also plays an important role in functionality of polymers for NA delivery both in vitro and in vivo. For instance, in a systematic study, a library of 480 block copolymers comprising of different amino thiol and alkyl thiol functional groups, with different ratios, was screened for in vitro mRNA delivery to cancer cell line IGROV1. A high‐throughput screen of this library of polyesters led to the selection of a few chemistries that were further formulated with Pluronic F127 to increase stability for subsequent in vivo studies. While in most cases the addition of F127 came at the cost of low transfection efficiency, reducing its content to 5% F127 was identified as the suitable condition for effective in vivo delivery and expression of mRNA in the lungs when administered intravenously to mice.^[^ ^139^ ^]^

***v) Polymer chain rigidity*** affects not only the binding efficiency/interaction with NA payload, but also the eventual biological functions such as endosomal escape^[^ ^135^ ^]^ and thereby transfection efficiency. Likewise, rigidity of nanoparticles, which can be a function of the T_g_ of respective polymers can influence both efficiency and the pathway of cellular uptake.^[^ ^140^ ^]^ Despite many studies investigating the role of resulting NPs rigidity, findings are in some cases controversial. For instance, while some studies reported higher cellular uptake for rigid NPs,^[^ ^141^, ^142^, ^143^ ^]^ others reported conflicting results.^[^ ^140^, ^144^ ^]^ Simulation studies correlated the rigidity of NPs with the energy barrier they have to overcome for internalization through membranes.^[^ ^145^ ^]^

In a comparative study, Miyazaki and colleagues evaluated mRNA delivery using two different types of cationic block copolymers, namely poly(ethylene glycol)‐poly(glycidylbutylamine) (PEG‐PGBA) and PEG‐poly(L‐lysine) (PEG‐PLL), with different chain rigidity. They found that PEG‐PGBA with more flexible polyether backbone resulted in formation of polyplexes with 50‐fold stronger interaction with mRNA molecules than PEG‐PLL comprising rigid peptide bonds. The augmented binding of mRNA to PEG‐PGBA led to the improvement in biological performance of polyplexes, such as protection of NA payload from enzymatic degradation, protein overexpression, and bioavailability, compared with PEG‐PLL, evaluated both in vitro, and in vivo.^[^ ^146^ ^]^

Gene transfection is a succession of discrete processes, i.e., cell interaction, internalization, release of genetic material among others, each of which places different demands on the delivery system.^[^ ^147^ ^]^ More detailed investigation of, e.g., cellular uptake and co‐localization can help to elucidate the contribution of polymers to overcome such bottlenecks, in order to optimize transfection beyond the often‐exclusive focus on the prototypical readout “transfection efficiency (%)”.

### 2.3 Tertiary Level: Complexation

While the complexation between polymeric carriers and NAs relies on weak intermolecular interactions, the resulting polyplexes exhibit properties distinct from those of the individual components. Indeed, these features are in another level of structural organization, which we refer to as the tertiary level, as it deals with another level of complexity. Size, shape, distribution of particles, surface charge, colloidal stability, stimuli‐responsive release, degradation, and additional surface conjugations are pronounced features of polyplexes, that are best considered for these entities rather than the individual components.

#### 2.3.1 Size

A carrier's physicochemical properties, such as ionization level, charge density and, in case of block copolymers, the ratio of nonionic to cationic blocks determine the type and extent of interaction with payload and eventual size of the polyplexes. When homopolymers or random copolymers are used, polyplex size is determined by the number of macromolecules contributing to the formation of a polyplex (as a function of preparation method), but also by the strength of interaction. Polymers with low charge density tend to form large, gel‐like polyplexes, whereas highly cationic macromolecules result in the formulation of compact NPs.^[^ ^136^ ^]^

When block copolymers are used for condensation, so‐called micelleplexes can form, where the interelectrolyte complex is surrounded by a corona of polymer arms, based on the noncationic block of the polymer. This also restricts the size of individual polyplexes and increases colloidal stability, i.e., stability of NPs size, charge, and dispersion in physiological environments, if the corona is highly hydrated. Amphiphilic diblock copolymers made of a cationic block, i.e., poly(*N*‐(2‐aminoethyl) methacrylamide) (PAEMA) and a nonionic block, i.e., poly(2‐deoxy‐2‐methacrylamido glucopyranose) (PMAG) with two different degrees of polymerization, i.e., PMAG_56_‐b‐PAEMA_30_ and PMAG_52_‐b‐PAEMA_63_ were tested in parallel with the corresponding homopolymers. Polymers with less cationic content resulted in the formation of less compact but more stable NPs. The cationic homopolymer, with higher positive charge density compared to diblock co‐polymers, initially led to the formation of smaller polyplexes. However, the resulting NPs tend to aggregate, thereby forming larger polyplexes. In contrast, nonionic blocks in diblock copolymers hinder formation of such aggregates and thus the eventual particle size was consistently smaller (**Figure** **6**a).^[^ ^148^ ^]^

The size of the polyplexes can affect cellular uptake mechanism and subsequent intracellular trafficking of NPs.^[^ ^145^, ^149^, ^150^ ^]^ Indeed, there is a lack of consensus in how size affects the cellular uptake and NA delivery. For instance, by screening a range of fluorescent particles with defined size from 50 to 1000 nm, it was reported that microspheres with a diameter smaller than 200 nm were taken up by clathrin‐coated pits. However, by increase in size of particles, caveolae‐mediated uptake was the predominant internalization pathway.^[^ ^151^ ^]^ Size‐dependent uptake route of DNA complexed with glycosylated polylysine or polyethylenimine was evaluated by studying three size ranges. While small polyplexes (<100 nm) were reported to internalize through caveolae‐dependent pathways, intermediate (100‐200 nm) and large polyplexes (>200 nm) were found to enter cells via clathrin‐coated pits, and macropinocytosis, respectively.^[^ ^152^ ^]^ In a comparative study, size‐dependent endocytosis of carboxylated polystyrene (PS) NPs of 40 and 150 nm diameter, considered as small and large size NPs for transfection, were comprehensively evaluated by confocal microscopy and single‐particle tracking method. Clathrin‐dependent endocytosis was the major pathway by which NPs with smaller size entered the cells, whereas larger NPs were taken up by cells through caveolae‐mediated endocytosis. Interestingly, upon internalization, large NPs tend to recycle back via exocytosis, suggesting the potential effect of NPs size on cellular retention of polyplexes.^[^ ^153^ ^]^ Particle size can also determine cellular uptake mechanisms. For instance, particles larger than 200 nm are typically taken up via phagocytosis. Leveraging this size‐dependent uptake mechanism, a recent study demonstrated ligand‐free targeting of monocytes and macrophages with PBAE/mRNA submicron particles formed via controlled supramolecular assembly.^[^ ^154^ ^]^ The lack of consensus in size‐dependence of the uptake pathway needs to be further pursued by high throughput screening of libraries of polyplexes with various sizes, ideally in different in vitro systems.

Particle size can also affect biodistribution when administrated in vivo.^[^ ^155^ ^]^ A systematic study of amine‐modified silica NPs with different sizes, ranging from 125 to 570 nm, revealed a direct correlation between cellular uptake and particle size, whereas DNA binding affinity was consistently reduced with increasing particles size. Consequently, the highest transgene expression was observed for NPs with medium size (330 nm).^[^ ^156^ ^]^

A straightforward tool to determine polyplex size is dynamic light scattering (DLS), which enables rapid screening of, e.g., different polymer/NA ratios. Multiangle light scattering, alone or coupled to a chromatography setup like asymmetric flow field flow fractionation^[^ ^157^ ^]^ enables much more detailed insights into particle size among other properties but is also connected to much greater analytical effort. Microscopic techniques like atom‐force microscopy (AFM) or scanning electron microscopy (SEM) offer a complementary view and, coupled with image analysis software, can be used to determine size and distribution. It should be noted however, that for both techniques dried, deposited samples are used, which can falsify the real size/morphology/state of aggregation of the sample as a result of sample preparation. In cryo‐transmission electron microscopy (cryoTEM) this effect can be partially mitigated.

#### 2.3.2 Particles Size Distribution

Size dispersity of polyplexes, commonly referred to as polydispersity index (PDI) can influence biological activity. When aiming to deduce structure–property relationships, low PDIs are preferred. Size distribution of polyplexes can be determined via process parameters. One such example is the mixing technique applied in complex formation, which can be performed either via conventional batch reactor/manual mixing or by a microfluidic mixing device. This is of particular interest for micelleplexes, where bulk mixing of polymer and NA solutions result in high complex dispersity due to the undefined nature of the actual self‐assembly process. For instance, in a comparative study, micelleplexes made of triblock copolymer polyethylenimine‐polycaprolactone‐polyethylene glycol (PEI‐PCL‐PEG) were prepared via the two mentioned methods for siRNA delivery. However, the microfluidic method resulted in more uniform particle formation with an average smaller size compared with the bulk mixing method. While no significant difference were reported for overall in vitro performance of the two approaches, neither in terms of endocytic pathway, and endosomal release nor eventual knock‐down efficiency, in vivo delivery was substantially improved for polyplexes prepared via microfluidics.^[^ ^158^ ^]^ On the contrary, another report using bioreducible linear poly(amido amine) polymers complexed with either pDNA or mRNA via bulk or microfluidic assisted confinement, suggested that the microfluidic preparation of polyplexes also improved in vitro NA delivery.^[^ ^159^ ^]^

Payload conformation/secondary structure can also affect particle size distribution, by influencing the interaction with polymeric carrier chains. In one study, complexation of pDNA versus linear DNA of the same construct with a diblock copolymer, poly(2‐deoxy‐2‐methacrylamido glucopyranose)‐block‐poly(N‐(2‐aminoethyl) methacrylamide) (PMAG‐b‐PAEMA) were examined in a side‐by‐side experiment. A bimodal size distribution was consistently observed for polyplexes containing linear DNA, whereas pDNA resulted in the formation of polyplexes with smaller size and a narrower size distribution, when screened for different ratios of polymer and NA. The latter was correlated to condensed conformation of supercoiled pDNA, which provides restricted interaction with the polyanionic chains of the carrier, compared to linear DNA, which can more readily interact with the carrier due to its extended molecular conformation.^[^ ^148^ ^]^

Like for size, DLS gives a useful readout for size distribution. Here, differentiation between the applied modes of data treatment is important to consider: while intensity weighted distribution is more likely to show larger assemblies or aggregates (as their scattering intensity is much higher), a number weighted distribution will emphasize smaller particles (that are usually present in larger numbers). Microscopy analysis coupled with software supported counting and measurement will also reveal distributions of particles size.^[^ ^160^ ^]^ However, reliable data require a large sample size.

#### 2.3.3 Shape

**Figure 5:**
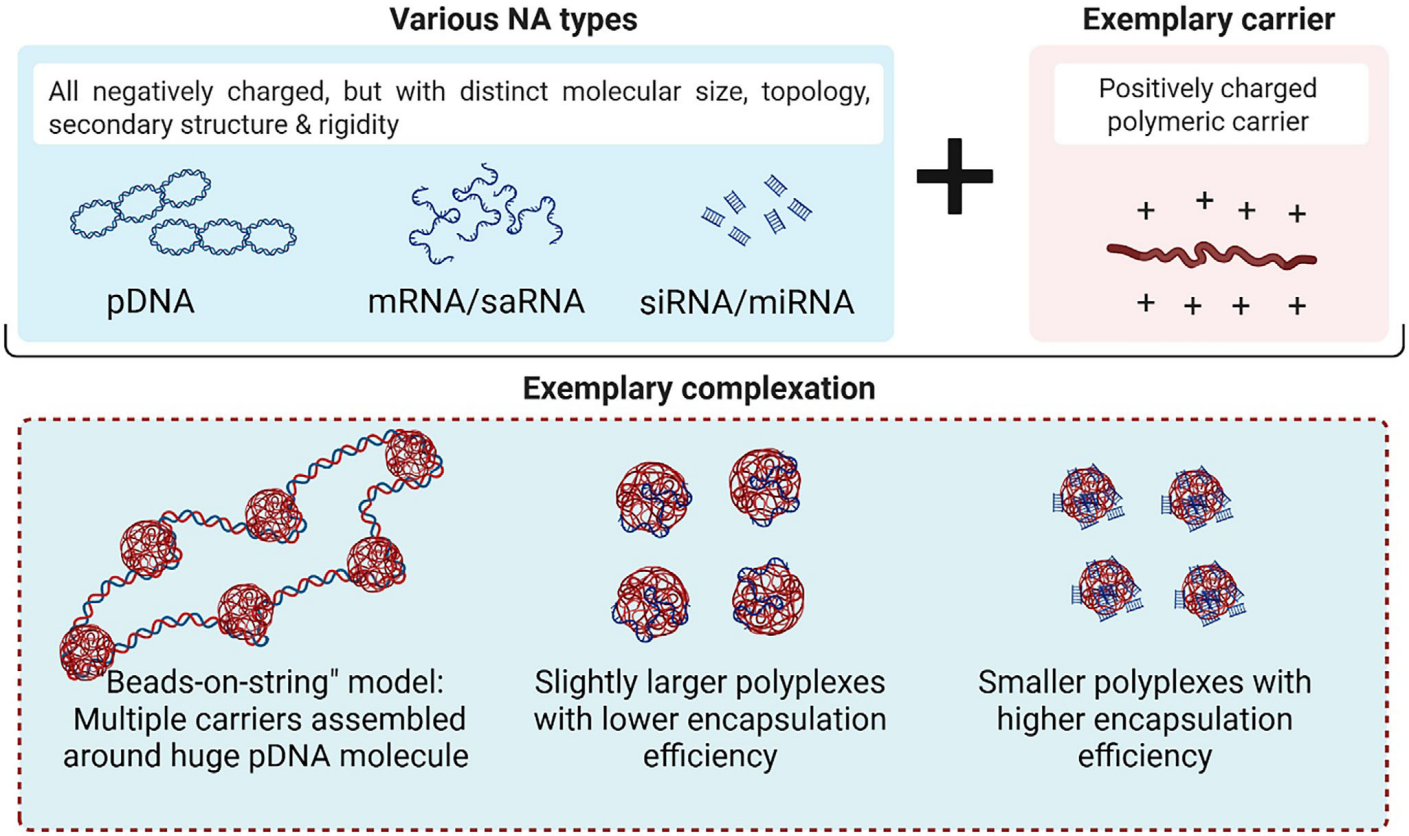
Effect of various types of payloads with distinct properties on subsequent complexation mechanism and shape of polyplexes. Illustrated on the lower panel is the exemplary polyplex formation. Beads‐on‐string model adapted from Ref. [161] (Created with BioRender.com).

**Figure 6:**
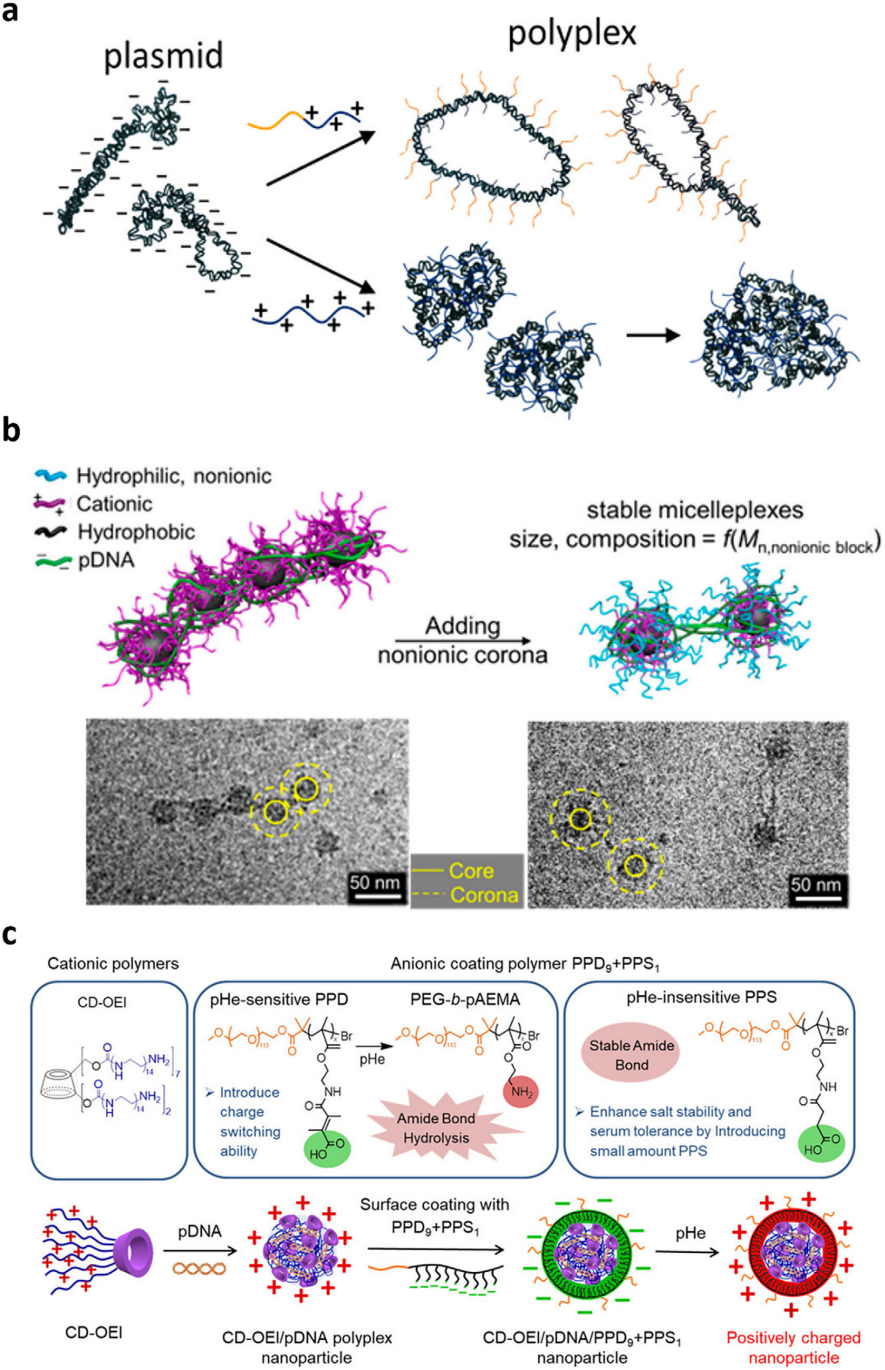
Different perspective indicated in size, shape and surface of polyplexes: a) Effect of different types of polymeric carriers, i.e., hydrophobic as well as cationic homopolymers, versus block copolymers with different size ratio of the cationic to hydrophobic blocks on the overall conformation and size of polyplexes. Reproduced with permission.^[^ ^182^ ^]^ Copyright 2017, American Chemical Society. b) “Beads‐on‐string” conformation of pDNA complexed with various block copolymers, indicating lower numbers of micelles (beads) on micelleplexes made of copolymers with larger nonionic segment. Reproduced with permission.^[^ ^161^ ^]^ Copyright 2018, American Chemical Society. c) Surface charge switchable polyplexes designed for active pDNA delivery to tumor cells with characteristic acidic pH Reproduced with permission.^[^ ^172^ ^]^ Copyright 2020, American Chemical Society.

The shape of polyplexes is determined by the size and conformation of both carrier and payload, as well as their subsequent interactions. For instance, polymeric micelles with different hydrodynamic radii and core sizes, made of block copolymers with different molecular weights, were reported to compact large pDNA molecules into chromatin‐like “beads‐on‐string” structures (**Figure** **5**). The number of micelles per micelleplex, i.e., string, decreased with increasing molecular weight of the nonionic segment (Figure 6b).^[^ ^161^ ^]^ In another study, PEI‐based NPs, either randomly assembled without controlled complex morphology or PEI‐coated spherical NAs containing radially oriented siRNA, were investigated side by side. The spherical polyplexes were found to be 10‐fold more efficient in gene silencing compared to randomly assembled polyplexes, while showing faster cellular uptake and lower cytotoxicity, as evaluated in U87‐MG, and U373 glioma cells and 293TN cell lines.^[^ ^162^ ^]^

The shape of polyplexes on the other hand will affect their rate and mechanism of cellular uptake. Various shapes such as sphere, rods, discs and cubes featuring different aspect ratios correlate with uptake efficiency.^[^ ^163^, ^164^ ^]^ For example, the highest cellular internalization was reported for spheres which have a lower aspect ratio compared with cubes and rods.^[^ ^165^ ^]^ When bottle brush copolymers are used to condense NA, resulting polyplexes can possess an elongated shape.^[^ ^137^, ^166^ ^]^

The higher aspect ratio was reported to decrease the cellular internalization rate, while uptake mechanism, intracellular trafficking and unpacking rate of the DNA payload were not influenced. The rod‐like particles resulted in delayed delivery as they were mainly accumulated in endosomal or lysosomal compartments.^[^ ^166^ ^]^

#### 2.3.4 Surface Charge

The surface charge of polyplexes can impact on extracellular stability, uptake mechanisms and subsequent endosomal escape,^[^ ^122^ ^]^ as well as their stability in blood circulation during in vivo delivery.^[^ ^155^ ^]^ Zeta potential measurements are the standard method for evaluating the charge density of polyplexes and are most often reported to remain positive even after complexation of polymeric carriers with anionic NAs. Micellar polyplexes can be formed using multiblock copolymers, with each block designed to fulfill a specific function. A showcase is an ABC‐type polypept(o)ide‐based triblock copolymer, comprising a shielding block (A), a bioreversible crosslinking block (B), and a cationic polypeptide block (C), forming a polyion complex micelles (PIC) for siRNA delivery.^[^ ^167^ ^]^ The overall positive charge of polyplexes is beneficial for promoting endosomal uptake via their electrostatic absorption and thus temporary disruption of the anionic cell membrane.^[^ ^163^ ^]^ However, excessive positive charge can also lead to unwanted interactions with anionic serum proteins, potentially resulting in variable transfection outcomes depending on the target cell type. Adsorption of serum proteins onto the polyplex surface can induce aggregation thereby preventing polyplexes from reaching to their target cells. This is particularly important for in vivo experiments, where serum cannot be excluded from the microenvironment. Shielding the polyplex surface with hydrophilic PEG moieties, commonly known as PEGylation, is a prominent approach to enhance NPs persistence in the circulation system in vivo and enhance biocompatibility by covering extra positive surface charge. However, this comes at the cost of lower transfection efficiency, due to reduced cellular uptake and transgene expression.^[^ ^168^ ^]^ Copolymers comprising the poly((*N*‐acryloylmorpholine) (PNAM) as one block were recently proposed as an alternative to PEG, effectively shielding cationic‐hydrophobic multicomponent polymer system complexed with pDNA.^[^ ^169^ ^]^

Shielding can also be achieved using anionic polymers that cover the surface of positively charged core polyplexes. For instance, anionic polymers such as poly(acrylic acid) (PAA and PNAM were applied to the surface of cationic micelles assembled from pDNA and poly[(n‐butyl acrylate)‐b‐(2‐(dimethyl amino)ethyl acrylamide)] (P(nBA‐b‐DMAEAm)) block co‐polymers. It was reported that compared to cationic micelleplexes, the anionic polymer shielding resulted in improved cell viability without compromising the transfection efficiency.^[^ ^170^ ^]^

New formulations have been developed in a way that the positive charge will be hidden within the inner core of polyplexes, supporting NA packaging, with the outer layer being either hydrophobic or negatively charged to avoid undesired interaction with serum.^[^ ^171^ ^]^

Surface charge can also be rationally designed to dynamically change in response to microenvironmental cues such as pH, thereby conferring different properties, necessary to accommodate different functions. In a recent study, surface charge‐switchable polymeric NPs were synthesized for DNA delivery into tumor cells, which typically exhibit more acidic milieu (Figure 6). A cationic polymer/DNA polyplex with a positive surface charge was coated with a pH‐sensitive block copolymer, composed of poly(ethylene glycol) (PEG), poly(2‐aminoethyl methacrylate) (pAEMA) conjugated to 2,3‐dimethylmaleic anhydride (PPD). The resulting double‐layered NPs exhibited a negative surface charge at physiological pH 7.4, which switched to positive charge in acidic pH (<6.8). The former property shielded polyplexes and increased their serum stability, while the latter enhanced cellular uptake, resulting in selective transfection of tumor cells (Figure 6c).^[^ ^172^ ^]^

#### 2.3.5 Polyplex Stability

Stability is another important factor which refers to the ability of a cationic carrier to maintain the integrity of polyplexes in the presence of other competing negatively charged molecules in extracellular milieu, an important factor for NA delivery systems^[^ ^173^ ^]^ Heparin as an anionic competitor is a commonly used biomolecule for modeling such situations and evaluating the potential release of the payload. If colloidal stability is low, so is the efficiency of the polyplex in protecting, transporting and releasing its NA payload. But also, colloidal stability is important, as polyplexes that aggregate and sediment fast, for instance in the presence of serum proteins, are of little use in transfection applications. Physicochemical properties of the polymeric carrier such as molar mass, and polymer structure determine stability of the polyplexes, whereas shielding segments like for instance the corona in micelleplexes can enhance colloidal stability drastically. For instance, the effect of molar mass of an outer nonionic PEG block of an amphiphilic tri‐block co‐polymer (PEG‐b‐PDMAEMA‐b‐PnBMA) was systematically investigated and found to be directly correlated with colloidal stability of micelleplexes for pDNA delivery.^[^ ^161^ ^]^ Despite being essential for protection of NA from degradation, too much stability leads to a decrease in transfection efficiency, by hampering intracellular polyplex unpacking and subsequent release of NA payload. In this regard, a copolymer made of quinine and 2‐hydroxylethyl acrylate was examined for pDNA delivery in vitro. Stable polyplexes were formed by combination of electrostatic interaction and intercalation of quinine within pDNA molecules. In the presence of intracellular proteins, however, polyplexes were less stable and released their pDNA payload, which was validated by endogenous tracking of quinine via Raman chemical imaging.^[^ ^174^ ^]^

#### 2.3.6 Stimuli Responsiveness

An attractive approach for developing “smart” carrier systems is to incorporate stimuli‐responsiveness, enabling controlled release or enhanced trafficking of NAs. Stimuli can be broadly classified as external triggers (light irradiation, hyperthermia, ultrasound) or endogenous microenvironmental cues (pH, redox, ATP). Importantly, the effect of a stimulus depends on the stage of delivery at which it is applied, such as polyplex stability, intracellular trafficking, or payload release.

- **Endosomal escape (pH‐ or light responsive)**

pH‐responsiveness is frequently exploited to facilitate endosomal release. A recent example is a charge‐altering β‐amido carbonate‐based polymer that undergoes nitrogen‐to‐oxygen acyl shift at acidic pH, resulting in charge cancellation and efficient mRNA release. This carrier enabled robust transfection of primary T cells in vitro and selective expression in the spleen in vivo.^[^ ^175^ ^]^ External triggers can also be applied: photochemical internalization (PCI) induces local ROS formation upon light irradiation, destabilizing endosomal membranes. Using this principle, light‐assisted delivery of DNA with polypeptides produced by ring‐opening polymerization achieved nearly 90% endosomal release, far exceeding PEI‐mediated transfection.^[^ ^176^ ^]^

- **Cytosolic release (redox‐ or ROS responsive)**

Redox‐sensitive carriers exploit the cytoplasmic abundance of glutathione, which is up to three orders of magnitude higher than extracellular levels.^[^ ^177^, ^178^, ^179^ ^]^ For example, disulfide‐crosslinked PEI derivatives showed improved unpacking of pDNA and higher transfection efficiency than their noncrosslinked counterparts.^[^ ^180^ ^]^ Similarly, ROS‐degradable PEI‐based nanoparticles enhanced pDNA delivery while reducing cytotoxicity compared with PEI25k, both in vitro and in vivo.^[^ ^181^ ^]^

- **Targeted delivery and accumulation (ATP responsive)**

Other stimuli‐responsive systems aim at cell‐ or tissue‐specific release. For instance, an ATP‐responsive ternary polyplex coated with recombinant high‐density lipoprotein selectively delivered siRNA to CD36⁺ macrophages in atherosclerotic plaques in vivo. Interestingly, cellular uptake increased CD36 expression, creating a positive feedback loop that promoted progressive nanoparticle accumulation and ultimately induced plaque regression.^[^ ^102^ ^]^

#### 2.3.7 Degradation

Degradation of polyplexes can also affect functions such as payload release and can be implemented by the cleavage of the carrier polymer backbone. Hydrolytic and enzymatic degradation are the main scenarios, which cover the fate of the majority of NA delivery carrier systems. Addition of ester bonds to nondegradable polymers such as PEI have been used to reduce its toxicity upon degradation.^[^ ^183^ ^]^ Polymers comprising urethane, imine, ester and orthoesters groups are other examples of hydrolyzable polymers. One prominent example is recently developed depsipeptide‐based copolymers, which contain both amide and ester groups.^[^ ^184^ ^]^ Upon hydrolytic degradation, they decompose to L‐amino acids units, which are capable of buffering pH, and protecting the degradation microenvironment. This avoids potential inflammation in acidic environment, which often occurs as a result of aliphatic polyesters degradation.

Another example is the use of noncationic polymers such as PLGA, for NA delivery, owing to their degradability, as well as lack of toxicity of degradation products. Instead of electrostatic interaction, NA was encapsulated within PLGA NPs by the double emulsion method.^[^ ^185^ ^]^ Addition of matrix metalloprotease (MMP)‐cleavable peptide linkage is an option when designing a degradable carrier system susceptible to enzymatic degradation.^[^ ^186^, ^187^ ^]^ Of note, the feature of degradability might also cause extra challenges in cytotoxicity, as not only the polymer itself but also its degradation products have to be considered.

#### 2.3.8 Addition of Cell or Organ‐Specific Targeting Ligands

Most therapeutic applications using NA‐based nanomedicine require delivery to a particular organ or ideally a specific cell type. However, the majority, if not all NPs are cleared by sinusoidal endothelial cells in liver shortly after in vivo administration. To inhibit this unintended early clearance and support the biodistribution to the desired target tissue/organ, NPs can be coated with a hydrophilic moiety, e.g. PEG, which avoids unspecific adsorption of proteins onto the surface of NPs and thus increases their blood circulation, also known as “stealth coating.”^[^ ^188^, ^189^ ^]^ The problem is that in many cases these approaches interfere with the intended function and universally decrease cellular uptake, which is not ideal for NA delivery. Still, the cloaking of carriers in targeting applications is essential, since for specific interactions to dominate, unspecific ones have to be reduced to a minimum. In an alternative stealth approach, Kataoka and colleagues suggested co‐administration of two‐armed PEG‐conjugated oligo(L‐lysine) with NPs. While PEG‐OligoLys selectively adhere and mask liver sinusoids, the NPs have a chance to access unmasked endothelium of other tissues.^[^ ^189^ ^]^

Another common approach for targeting a specific cell‐type is the addition of a surface ligand,^[^ ^190^ ^]^ that promotes selective uptake only by specific cell type with a ligand‐corresponding receptor in vitro, or organ‐specific localization of NPs in vivo. For instance, addition of mannose to the surface of polyplexes was reported to increase specific uptake by macrophages^[^ ^191^ ^]^ or dendritic cells.^[^ ^192^ ^]^ Folic acid has also been implemented by numerous studies for targeting cancer cells.^[^ ^193^ ^]^

Amphipathic peptides, e.g. trans‐activating transcription factor (TAT) derived from HIV virus, or melittin from honeybees,^[^ ^194^ ^]^ could be designed to either promote uptake or facilitate endosomal escape by being selectively displayed at the surface of polyplexes in response to pH decrease in late endosomes.^[^ ^195^ ^]^ For instance, Pan et al. evaluated the uptake pathway of a rationally designed cell penetrating peptide. As cellular uptake could not be blocked by inhibiting the known endocytosis pathways, they excluded endocytosis as a cellular uptake/trafficking pathway. They further supported this assumption by fluorescent labeling of NA and live tracking of polyplexes via time‐lapse confocal microscopy, where they observed no co‐localization of endosome/lysosome with siRNA polyplexes. Besides, by observing an ATP‐independent uptake of NPs, they have suggested direct cellular translocation as a potential entry pathway.^[^ ^196^ ^]^

### 2.4 Quaternary Level: Integration Within 3D Structures

In the next level of organization, polyplexes can be incorporated within 3D structures such as scaffolds, hydrogels, and microparticles. This serves purposes like: i) producing an in vitro model that can more precisely mimic the ultimate in vivo 3D microenvironment of cells than of ordinary 2D cell culture systems, ii) increasing stability and thus life span of carrier systems by shielding/protecting of polyplexes, iii) achieving local NA delivery for in vivo applications, which results in a higher target cell transfection versus nontarget cells when compared to systemic delivery as well as iv) providing sustained release conditions. Ideally, incorporation of NPs within a 3D structure supports temporal and spatial modulation of polyplex delivery and bioresponsive release in more advanced applications. In most cases, the basic principle of polyplex integration with 3D structures mentioned above is physical entrapment within polymeric matrix structures, often also referred to as encapsulation.^[^ ^197^, ^198^ ^]^

Tissue‐like water content, injectability, tunable physicochemical and biological properties make hydrogels appealing candidates for tissue regeneration studies. Embedding polyplexes within hydrogel meshes was initially introduced as an add‐on feature which improved the overall biological function of hydrogels without a major impact on their physical (e.g., mechanical) properties.^[^ ^199^, ^200^ ^]^ Initial attempts were made by embedding PEI‐pDNA polyplexes within PEG‐based hydrogels, which were further refined with MMP cleavable crosslinks. Upon cell migration toward the hydrogel core, and concomitant production of MMP, hydrogel meshes could be degraded and polyplexes released.^[^ ^201^ ^]^ Hydrogel structures have also been designed to support in situ gelation in response to specific physiological pH and temperature.^[^ ^202^ ^]^ Combination of PBAE/mRNA nanoparticles with a thermo‐responsive block copolymer has been recently reported to enable local retention and efficient transfection of cells at a tumor injection site by gelation of the mixture upon injection. Co‐delivery of mRNA encoding immunostimulatory cytokines led to the recruitment of immune cells and induced immunological reprogramming at the tumor site.^[^ ^203^ ^]^

Polyplexes have also been integrated within microparticulate systems, so called “Nanoparticles‐in‐Microparticles delivery system.” For instance, polyplexes were entrapped within a core layer of PLGA microspheres formed via a double‐emulation method.^[^ ^197^ ^]^ These microparticles were designed such that they release their core payload, the polyplexes, in response to defined microenvironmental changes. This created an advanced multistep delivery system able to support and release the payloads at different stages of extra‐/intracellular delivery pathway.^[^ ^204^ ^]^

Further, the positive surface charge can be actively harnessed to facilitate polyplex interaction with an adjacent substrate, e.g. the surface of microparticles or hydrogels, constituting of building blocks of a higher ordered structure. This is applicable for the sequestered/prolonged release of protein products that are initially entrapped within the matrix upon secretion from transfected cells. This might be an alternative approach to sustained release of NA payloads. The extended effect is particularly advantageous for NA entities with highly transient expression such as mRNA. Such an approach was recently implemented by Murphy and colleagues, where mRNA loaded NPs were electrostatically adsorbed on the surface of inorganic microparticles, resulted in biomaterial‐mediated sequestration of overexpressed protein.^[^ ^205^ ^]^

Solid scaffolds such as electro‐spun nanofibers, membranes or sponges also share the capacity for conjugation with polyplexes through physical/chemical interaction.^[^ ^206^ ^]^ Traditionally named “gene activated matrixes” (GAMs),^[^ ^207^ ^]^ and more recently, upon development of in vitro transcribed mRNA in NA delivery field, “transcript activated matrixes”(TAMs)^[^ ^208^, ^209^ ^]^ are examples of scaffold mediated release of polyplexes. Despite initial studies introducing the concept of 3D transfection, further research is needed to achieve more robust and reproducible sustained release of polyplexes from 3D matrices.

## 3 Future Perspective

In this chapter, some emerging principles and ideas are presented, which might be more intensely followed in future studies to progress the “next generation” of smart, multifunctional polymeric carrier systems for NA delivery.

### 3.1 Complexity of Structure–Function Relation Network

**Figure 7:**
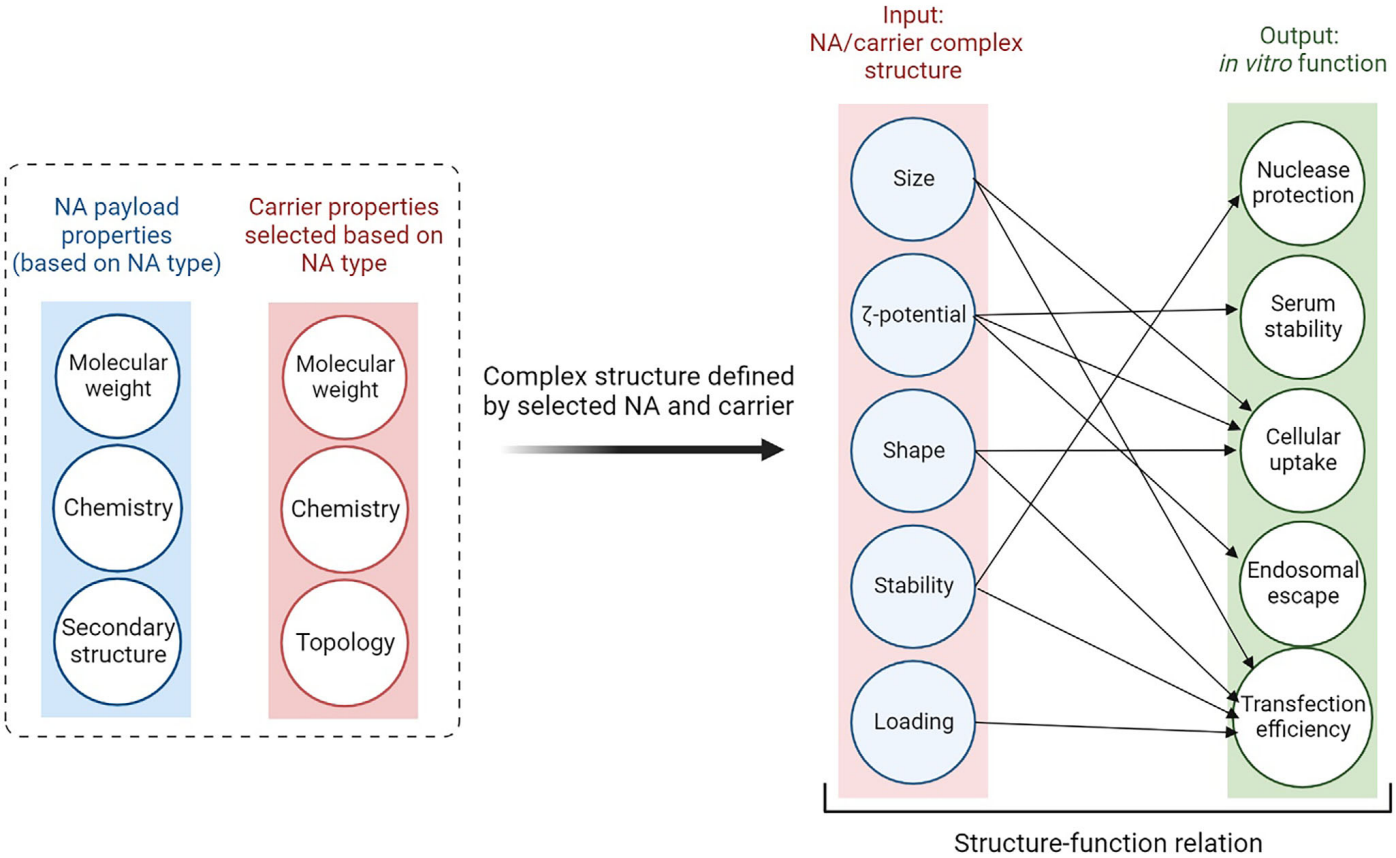
Complexity of crosstalk between multiple parameters indicating the structure‐function relationship for NA delivery inspired by neural network approach. This interconnected network emphasizes the essence of computer‐based modeling in future studies to solve and address this level of complexity (Created with BioRender.com).

Development of rationally designed NPs requires not only focus on individual physicochemical properties of a carrier or operating by trial and error, but also implementation of more integrative approaches like computational modeling techniques, e.g., neural network‐based models, for design of a carrier system with highly specific and predictable features suitable for a given application. The existing knowledge of carrier systems structural features and required functions, besides currently available data from previous studies, could be exploited to establish the connections and crosstalk within the complicated “*structure–function*” relationship network as an input for training of the computer model (**Figure** **7**). Consequently, this educated artificial system could provide the basis of new carrier design built on top of earlier knowledge, which would not be plausible otherwise. For instance, a state‐of‐the‐art machine learning algorithm was recently employed by Green and colleagues to make an in silico prediction of an ideal polymeric carrier for cell‐dependent DNA delivery. A dataset of PBAEs polymer properties was fed as input and transfection efficiency as well as toxicity in different cell types were defined as output to train several algorithms. The ability to perceive a structure‐function relationship and to predict DNA transfection were validated by experiments using RWA264.7 macrophage and Hep3B liver cancer cells.^[^ ^210^ ^]^

Harnessing computational models has the potential to facilitate cost‐effective discovery and production of highly customized delivery systems, in line with future demands of personalized medicine. In an early attempt, Bishop et al. implemented principal component analysis to elucidate the correlation between polymers selected physicochemical properties and three biological functions, i.e., transfection, uptake and viability. They further validated their model by transfecting human glioblastoma cells with a library of PBAEs with versatile features. Given that experimental data was consistent with what was predicted by modeling in terms of the most impactful physicochemical features on biological functions, such studies are pointing a way to a more predictive approach to carrier design.^[^ ^5^ ^]^

AI‐guided engineering of carrier systems was recently reported for development of a customized carrier system for mRNA delivery. Steps included design and synthesis of lipid libraries, in silico screening between over 10 000 candidates using neural networks by distinguishing various cell types from muscle cells to immune cells. The result suggested cell‐specific requirements regarding tail length and head groups of the lipids.^[^ ^211^ ^]^ While this study was performed on ionizable lipids for delivery, similar approaches can be employed for design of new polymeric carriers by combining deep learning and chemistry tools.

### 3.2 Dynamic Nature of Nucleic Acid Delivery Microenvironment

**Figure 8:**
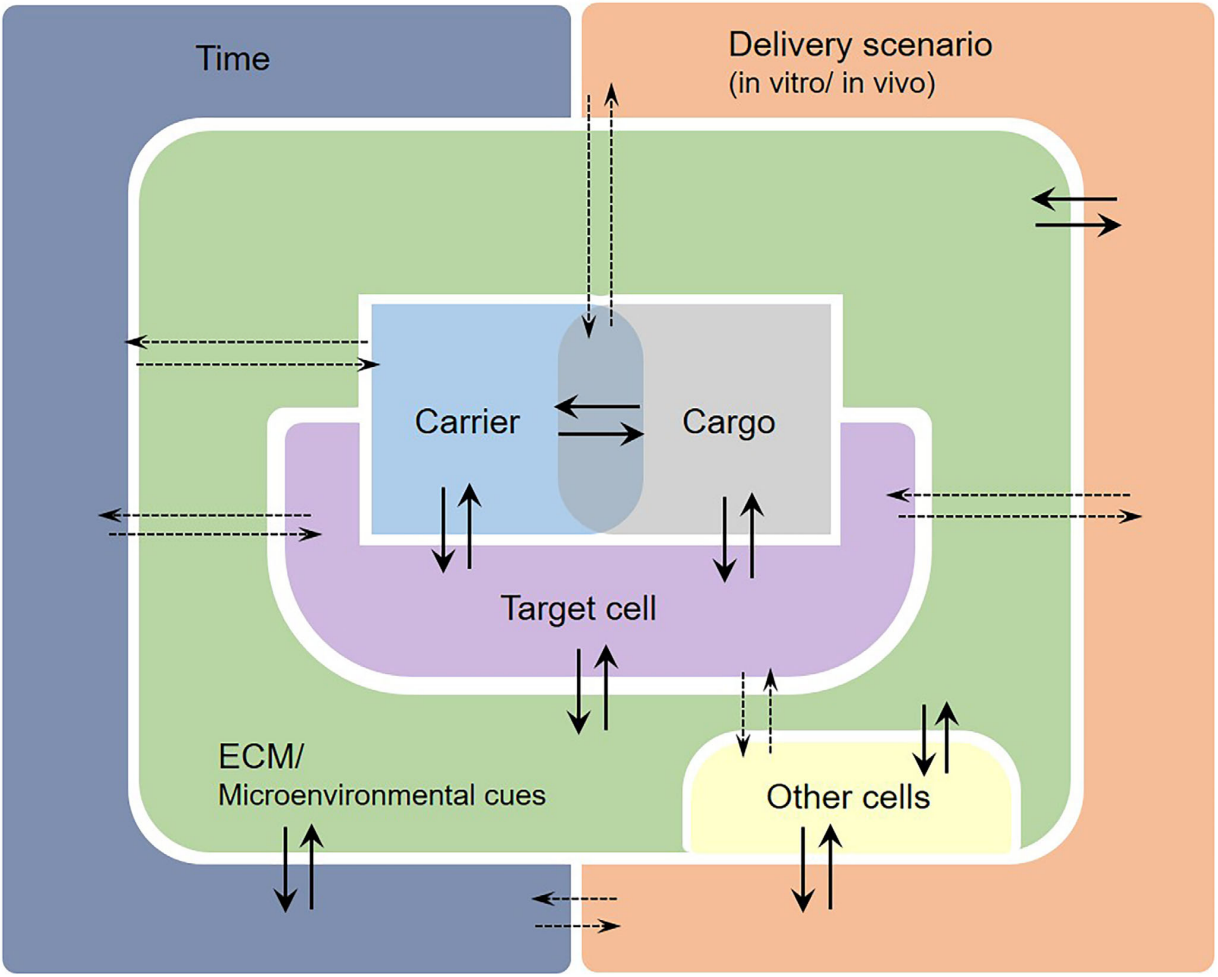
Dynamic nature of systems for NA delivery with regard to changes of each element's properties over time, and how these variations affect the rest of the systems. Double arrows indicate the reciprocal correlation of the two parameters. Dashed double arrows represent indirect impact of the elements on each other. These relationships and their dynamicity should be considered not only for rational design, but also in the analytical study of carrier system over time.

Constant variations in NA delivery microenvironments can affect and change not only carrier and payload properties, but also cellular microenvironments and target cell behavior. **Figure** **8** represents a simplified, schematized interconnection of various elements deemed essential for the success of NA delivery. Their variations are even more pronounced and likely less controllable in vivo. Therefore, being able to track those changes, and in more advance scenarios foresee and harness them in favor of accomplishing NA delivery would be of interest for future scientists and engineers. This inevitably would raise demand for robust quantitative technologies. For instance, spatiotemporal acquisition and analysis of data would be very beneficial for deeper understanding of trafficking processes.

### 3.3 Rational Design Versus Deep Learning Perspective

**Figure 9:**
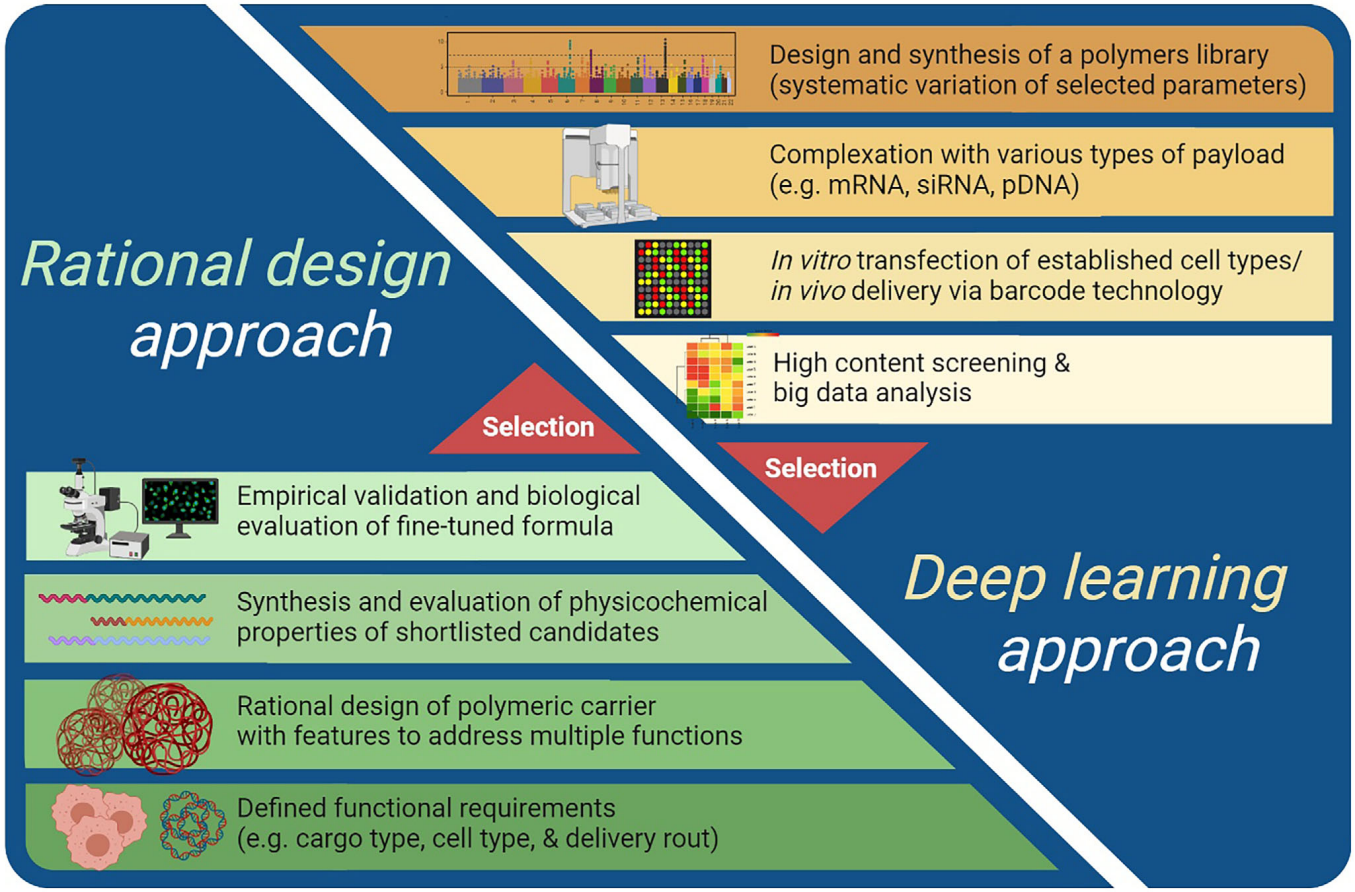
Two different approaches for developing new gene carrier systems. The deep learning approach involves using the library of existing polymers and finding the best fit to different payloads, whereas the rational design approach mainly deals with selection of a carrier system using platform advance technologies, which requires deep understanding of biological functions of the system. (Created with BioRender.com).

There are two principle approaches of finding new formulas for carriers in NA delivery (**Figure** **9**). The deep learning approach entails formation of a library of polymers with systematic variation of few parameters, such as chemical structure, molecular weight, etc., and subsequent high throughput screening and analysis, in order to find the best formulation for delivery of desired NA, e.g. pDNA, mRNA, and siRNA. Transfection could be initially done for established cell lines on top of the particular primary cell type of interest. One prominent example is 7C1 compound for in vivo siRNA delivery to endothelial cells. By screening a library of epoxide‐modified NPs, 7C1 was found to efficiently transfect endothelial cell in vitro and selectively in vivo.^[^ ^212^ ^]^ Green and colleagues established a machine learning pipeline for in silico NP screening for a library of biodegradable PBAEs for DNA delivery. The computational predictions were successfully validated in RAW264.7 macrophages and Hep3B liver cancer cell lines.^[^ ^210^ ^]^

To scale up the screening power in vivo, thousands of nanoparticles were evaluated simultaneously in the same animal model using DNA barcoding technology. These advances also have the potential to reduce the number of animals sacrificed for in vivo evaluation, while providing a massive amount of data/information about biodistribution and transfection of various formulas upon intravenous administration of carrier systems.^[^ ^213^ ^]^

The rational design approach, by contrast, mainly relies on rational and meticulous design of a carrier system to fulfil the specific functional requirements for the chosen payload and the delivery scenario. As such, rational design encompasses the synthesis of a few selected formulas and the empirical validation and selection of the final carrier. Although both methods involve substantial experimental efforts and both require high‐end technologies, once an effective, supporting computational model is established, the rational design approach can be applied in combination with the model and this could potentially result in a remarkably lower cost of production, a key concern for translational/clinical applications and upscale manufacturing within the biopharma industry.

### 3.4 Nanorobotic Delivery Systems

Nanorobotic systems represent promising and precise drug and potentially also NA delivery technologies, which release their payload in response to molecular triggers. In this sense, NAs could not only be the subject of studies as payload, as we discussed throughout this paper, but as a carrier for another payload such as drugs for cancer treatment. One fascinating category of such carriers are DNA origamis which are designed and constructed as programmed DNA nanorobots to transport their payload to specific locations in vivo such as to a tumor. In one study, targeting has been achieved by using a DNA aptamer against a specific receptor on cancer cells, while also mediating changes in DNA nanorobot structure from closed tubular to open sheet structure and subsequent release of its payload.^[^ ^214^ ^]^ Attempts to increase stability and delivery efficiency of such DNA carriers are in progress.^[^ ^215^ ^]^ For example, DNA origamis covered with cationic human serum albumin was reported to have resistance to nuclease‐mediated degradation, as well as enhanced cellular uptake.^[^ ^216^ ^]^ In summary, while a promising delivery technology, the nanorobot approach still awaits functional validation as an effective NA delivery tool. However, this is not only true for nanorobots, but in a broader perspective is also valid for all nanomedicine‐based approaches for NA delivery, referred to as “The beginning of the end of the nanomedicine hype.”^[^ ^217^ ^]^ This notion further emphasizes to focus on addressing functions and finding meaningful/deliberate solutions to reasonable problems with existing knowledge. This is in the contrary to the previous viewpoint to seek new and complicated (gene) delivery systems, which might not be necessarily as facile and applicable as they are theoretically predicted on paper.

### 3.5 Artificial Intelligence‐Based Approach for Payload‐Specific Polymeric Carrier Design

Artificial intelligence (AI) is playing a transformative role in fundamentally reshaping polymeric carrier design.^[^ ^218^ ^]^ An AI‐based approach leverages computational intelligence to streamline and optimize the design of payload‐specific polymeric carriers, tailoring them to the unique properties of individual NA entities.^[^ ^219^ ^]^ By integrating information about the NA payload such as molecular size, charge, solubility, and desired release kinetics with the intended biological target and pharmacokinetic profile, AI models can predict optimal polymer compositions and carrier architectures.^[^ ^210^ ^]^ These models are trained on large datasets of previously characterized carriers and NA payloads, enabling them to identify complex patterns and relationships that may not be immediately apparent through conventional design methods.

This approach enables the rapid generation and screening of candidate polymeric carriers, significantly reducing the time and resources required for experimental trial and error.^[^ ^220^ ^]^ Machine learning algorithms can suggest new material combinations, estimate their performance, and iteratively refine designs based on experimental feedback.^[^ ^221^, ^222^ ^]^ Notably, it is essential to develop new methodologies that enable fast synthesis of large polymer libraries to allow quick screening for validating and/or feeding computational models. For instance, a recent study developed a rapid synthesis and screening workflow using combinatorial blending of statistical copolymers to efficiently generate and evaluate a large polymer library, enabling accelerated identification of high‐performing, cell‐type‐specific carriers for pDNA delivery.^[^ ^223^ ^]^ However, not only synthesis but also generation of analytical and bioactivity data needs to be streamlined and automated to enable the generation of larger data sets, suitable for machine learning. Furthermore, AI tools support virtual simulations of carrier–payload interactions and in vivo behavior, providing early insights into efficacy and safety. A recent study showed that cationic micelles with diverse amine chemistries exhibit a strong correlation between in vitro delivery performance and in vivo lung‐targeted mRNA efficacy, underscoring the predictive value of in vitro models when paired with data‐driven nanoparticle optimization.^[^ ^224^ ^]^ Ultimately, this data‐driven methodology allows for the development of highly efficient, customizable polymeric carriers with improved delivery outcomes and accelerated translation to clinical application.

## 4 Conclusion

This review highlights the determining role of payload chemistry and structure in the rational design of a NA delivery carrier systems. Covering some basic definitions in genetic engineering and NA delivery, the structure‐function relationships of NA delivery carrier systems are elaborated. In order to deconvolute the complexity of delivery systems, we introduced a new organizational concept analogous to the four structural levels of proteins. In the first level, we addressed the chemistry of different NA entities as well as cationic polymers commonly employed for NA delivery. At the second level, the structural properties of payload and carriers were explained separately, regarding NAs as an anionic (bio)polymer component in the system. The complexation mechanisms and methods, together with the physicochemical properties of the resulting formulations and how they are influenced by the properties of their building blocks, were covered in the third level. Finally, to facilitate local as well as sustained release, polyplexes may optionally be incorporated into a 3D matrix, e.g. hydrogels or other biomaterial scaffolds, a concept that is relatively new in the field and requires further investigation and establishment.

Understanding all the aspects explained above is only the first step toward the rational design of carrier systems that best accommodate the requirements of a particular payload to be delivered. We underscore the differences between various types of NAs, such as plasmid DNA, double stranded linear DNA, single stranded mRNA, self‐amplifying RNA, small interfering (double stranded) RNA, etc. Notably, even in one given type of NA such as mRNA, there are potential variations in the 5′ end or in its nucleotide chemistry. While such chemical modifications are often incorporated for different purposes, e.g. to reduce immunogenicity, their influence on complexation with the carrier, and the resulting physicochemical properties of the polyplexes remains to be determined in future studies.

In order to fully move toward payload‐based rational design, more systematic high‐throughput experiments using existing advanced analytical tools are required. The resulting databases, comprising defined payload properties, carrier chemistries and systematically varied structural parameters, and the physicochemical properties of the resulting polyplexes, can then be used to train machine learning models, which can be the basis for the rational selection of design parameters in empirical studies. This approach accelerates advances in developing new formulations to meet emerging market demands, such as the pressing need to deliver vaccines in short periods of time. Overall, by shedding light on the complexity of the structure–function relationships in NA delivery systems, and underscoring the often‐overshadowed importance of payload properties, we provide guiding principles for the rational design of carriers and potential directions for future investigations.

## Conflict of Interest

The authors declare no conflict of interest.

## Supporting information



Supporting Information
