# Supplementary material for: Payload‐Driven Design of Polymeric Carriers for Nucleic Acid Delivery: Insights from Structure–Function Relationships
Source: Adv Sci (Weinh). 2025 Nov 29;13(4):e12653. doi: 10.1002/advs.202512653 (PMC12822472; doi:10.1002/advs.202512653)
Supplement: Supplementary file 1 — Supporting Information [file ADVS-13-e12653-s001.pdf]

Supplementary information:

Payload-Driven Design of Polymeric Carriers for Nucleic Acid Delivery: Insights from Structure–Function Relationships

Hanieh Moradian[1, 2,*], Charlotte Maeve Dunne[3], Manfred Gossen[1, 2], Matthias Hartlieb[4, 5]

1. Institute of Active Polymers, Helmholtz-Zentrum Hereon, 14513, Teltow, Germany
2. Berlin-Brandenburg Center for Regenerative Therapies (BCRT), 13353 Berlin, Germany
3. Pantherna Therapeutics GmbH, 16761 Hennigsdorf, Germany
4. Germany Institute of Chemistry, University of Potsdam, 14476, Potsdam, Germany
5. Fraunhofer Institute for Applied Polymer Research (IAP), D-14476, Potsdam, Germany

[*]Corresponding author: Hanieh Moradian, Helmholtz-Zentrum Hereon, Institute of Active Polymers, Kantstrasse 55, 14513 Teltow, Germany; Berlin-Brandenburg Center for Regenerative Therapies, Charité Campus Virchow-Klinikum, Föhrerstrasse 15, 13353 Berlin, Germany. hanieh.moradian@hereon.de

1 Genetic engineering: shuttling information into cells

The genetic information of eukaryotic cells is stored in their double-stranded, polymeric deoxyribonucleic acid (DNA), the DNA double helix. The two strands wind around each other are anti-parallel with respect to their 5' to 3' polarity and are organized in chromosomes as a molecular continuum. Genes are the principal functional unit of the stored information that is used, or in the language of biology "expressed", in a first step by the highly regulated process of transcribing DNA into chemically distinct, single-stranded ribonucleic acid (RNA) species. In the case of messenger RNAs (mRNA), this information is further passed on by the process of translation, resulting in the synthesis of proteins. This pathway of the flow of genetic information is often referred to as the "central dogma of molecular biology".[1] Aside from its expression, genetic information needs to be faithfully replicated, typically by the duplication of DNA, rarely RNA but never proteins. This process is further regulated by other non-coding RNA species, such as microRNAs.

The conventional way of adding to or perturbing the genetic information of higher eukaryotic cells via genetic engineering has been realized via the introduction of DNA, most often circular plasmid DNAs (pDNA). The purpose could be to gain biological insights by correlating genotypic changes with phenotypic outcomes, to produce recombinant proteins, or to enable therapeutic interventions. These exogenous pDNA molecules are typically equipped with transcription signals like promoters and enhancers upstream of the coding sequence for the gene under investigation and other processing signals. Upon cellular uptake, plasmid DNA (pDNA) can drive expression of a heterologous gene through transcription and subsequent translation of the encoded protein. Together, genetic information can be introduced into cells through biological, physical, or chemical methods, each employing distinct carriers and mechanisms of gene transfer.[2]

The biological approach relies on recombinants, i.e., genetically engineered viruses carrying the desired genetic information. These viruses infect target cells by exploiting naturally evolved mechanisms of viral entry. Depending on the type of virus, the delivered DNA can either integrate into the host genome—becoming heritable—or persist episomally, remaining outside the chromosome for varying durations. Some viruses, such as retroviruses, carry their genetic information in the form of RNA. In these cases, the RNA is reverse-transcribed into DNA upon cellular entry and can subsequently integrate into the host genome. The use of engineered viruses for nucleic acid transfer is broadly termed "transduction".[3]

In contrast, non-viral nucleic acid transfer by physical or chemical means is commonly referred to as "transfection". Typical physical approaches used for nucleic acid transfer rely on the formation of pores in the cell membrane, rendering it permeable for biomacromolecules like nucleic acids by either applying an electric field [4], exposing cells to ultrasound [5], or mechanically by squeezing cells

through microfluidic channels also known as mechanoporation.[6] Other techniques use biolistic, i.e., the delivery of nucleic acids via microprojectiles (a.k.a. *gene gun;* [7]) or direct microinjection of DNA or RNA solutions into the cell.[8]

The focus of this review is the other branch of transfection that by chemical approaches, particularly polymer-based approaches. Polymers can form submicron particles suitable as carriers that can be taken up by mammalian cells via various routes. When charged positively, such polymers have the intrinsic capacity to bind anionic nucleic acids via electrostatic interaction. Often this binding of nucleic acids is the prerequisite for particle formation toward a nanoparticle size-compatible for cellular uptake. When chosen as the basic building blocks for carrier systems for reasons other than their charge, such non-cationic polymers can be charge-functionalized to enable nucleic acids interaction [9]. In particular, polymeric NPs with multifunctional properties are not only beneficial for gene delivery but also for modification of cellular functions such as promoting proliferation, diminishing intracellular H_2O_2 or inducing apoptosis, which have been extensively reviewed elsewhere [10].

Non-polymeric carrier systems, which are beyond the scope of the current manuscript, include lipid-based carrier systems such as lipid nanoparticles (LNPs), liposomes or micelles [11]. Inorganic carrier systems, often implemented in hybrid forms, are nanoparticles or co-precipitates, most prominently calcium phosphate with nucleic acids[12].

2 Nucleic acid delivery in bioengineering and in therapeutic applications

This initial section of the review gives an overview of the main applications of genetic engineering via NAs transfection both in bioengineering and in therapeutic approaches. Given that recent progress in the field is mostly problem-focused or application-oriented, this short overview integrating both aspects should provide some guidance to researchers outside the biomedical and genetic engineering world by providing application-specific selection criteria for possible contributions using multifunctional carriers for gene delivery. Here we summarize and categorize the rationale behind genetic engineering in four of its major application areas, (i) upregulation of protein expression, (ii) knock-down of protein expression, (iii) nucleic acid vaccines, and (iv) genome engineering. In the following, each category is briefly defined and supported with relevant examples from the biomedical field.

2.1 Protein overexpression

Expression of a given protein can be transiently or permanently upregulated in a given eukaryotic cell via gene transfection in a "gain-of-function" approach. This strategy is pursued in basic biological studies, seeking to elucidate the function or action mechanism of a particular protein, such as enzymes [13], structural proteins [14], or secreted proteins like paracrine factors with their intercellular effect

on a specific signaling pathway [15]. Protein overexpression is also integral to therapy-oriented, biomedical research [16]. For instance, to elucidate whether a particular protein is responsible for a certain disease, or to determine if overexpression of a protein might have therapeutic relevance [17], studies can address these questions through protein upregulation *in vitro* as well as in *in vivo* animal models [18].

Aside from viral transduction, plasmid DNA (pDNA) and mRNA can be employed for protein overexpression (Fig. S1). However, due to their intrinsic differences in where and how the encoded genetic information is realized, pDNA and mRNA are often used for different applications. pDNA transfection can result in either transient [19] or, when chromosomally integrated, also permanent expression of the protein investigated or produced for biotechnological purposes [20]. For example, in a therapeutic research study, bone marrow mesenchymal stem cells (MSCs) were transfected with pDNA/jetPEI to transiently overexpress Bcl-2, an antiapoptotic gene, prior to injection into the dysfunctional left ventricle of a post-infarcted heart. The overall survival rate of transplanted cells was enhanced in Bcl-2 overexpressing MSCs, leading to a reduction in infarct size compared with non-transfected MSCs [21].

By contrast the use of mRNA will result in fast but only transient protein overexpression [16a]. Depending on the scientific question and its requirement, either of these nucleic acids can be selected. However, there are certain advantages to mRNA that make it an appealing alternative to pDNA, as discussed extensively in other reviews.[22]

2.2 Knock-down of protein expression

A common biological approach to identify an unknown protein's function is to inhibit its expression and subsequently examine the phenotype of cells lacking, or coping with reduced levels of that specific target protein. Such "loss-of-function" approaches have been pursued for many years, forming the basis of much of current biological knowledge. Protein down-regulation is also instrumental when several candidate malfunctioning proteins might be responsible for a certain disease. Here, transient down-regulation could be implemented, first, to clarify if that protein is correlated with the disease phenotype [23]; second, to use this as a potential therapeutic application. For example, studying a particular protein's role in tumor-cell development and inducing apoptosis by knocking down the corresponding proteins exemplify such approaches [24].

Post-transcriptional regulation mechanisms such as RNA interference (RNAi) process, which is a natural phenomenon occurring in eukaryotic cells, can be technically exploited to achieve target protein knock-down.[25] The RNAi process can be mediated by either small interfering RNA (siRNA), or micro RNA (miRNA), both are processed inside of the cell, where they bind to target proteins' mRNA and

result in its degradation or simply inhibit its translation, resulting in reduced protein levels, so-called "knock-down" of the protein (Fig. S1). However, there are differences between these approaches. SiRNAs are short synthetic double-stranded RNAs which have to reach the cytoplasm via a carrier, whereas natural miRNAs are endogenous single strand RNAs transcribed from the non-coding part of the genome for controlling gene expression. More detailed information about the structure of siRNA is elaborated in Section 2.1.1. of the main text, and the requirements for siRNA delivery including recent progress of nanoparticles in this field have been comprehensively covered in other reviews [26].

One example of a polymeric carrier that has advanced into clinical evaluation is STP705, a polyplex formulation of two siRNAs (targeting TGF-β1 and COX-2) with a Histidine-Lysine co-polymer (HKP) as carrier. The formulation is administered intradermally or intralesionally and was tested for treatment of cutaneous squamous cell carcinoma. In a recent Phase II trial, STP705 achieved histological clearance in ~76% of treated lesions, with favorable tolerability. Lack of toxicity supports the translational potential of polymer-based carriers for nucleic acid therapeutics.[27]

2.3 Nucleic acid vaccines and immunoengineering

There are two main technologies for gene delivery to immune cells, both developed for therapeutic applications: (i) NA vaccines, and (ii) immunoengineering. NA vaccine technology includes both DNA and mRNA-based formulations [28]. Compared with traditional vaccines, which are based on protein antigens, live attenuated pathogens or inactivated pathogens [28], this new generation of NA vaccines is potentially more cost-effective. Vaccination can be realized by DNA or mRNA–mediated expression of specific antigens in immune cells, e.g. antigen presenting cells (APCs) like dendritic cells (DCs) and macrophages (Mφs), to elicit immunity against the specific pathogen[29]. One prominent recent example of the former approach is expression of spike protein of SARS-CoV-2 virus which was subject to many studies as well as large pharmaceutical companies worldwide upon Covid-19 pandemic [30]. See [31] for more detail on pDNA-based vaccines,[32] for vaccines based on mRNA, and [33] for a more general overview of NA vaccines delivered via NPs.

The second application is immunoengineering, which deals with immune cell-based therapy for a wide range of diseases, such as cancer [29]. Target immune cells for gene delivery are APCs such as DCs, Mφs, as well as B- /T-lymphocytes. APCs are transfected either to express tumor antigens, or to secrete soluble signals, e.g. antitumor growth factors, to coordinate recruitment, expansion and activation of specific T-cell phenotypes to trigger, enhance or modulate immune responses [34]. Transfection of B-cells can also play an important role, because they help determine the subsequent T-cell response and overall immune reaction. Alternatively, T-cells can be directly transfected to express tumor-specific **c**himeric **a**ntigen **r**eceptors. These T-cells are referred to as "CAR-T-cells", and the respective

therapeutic approach as “adoptive T-cell therapy”. More detailed information about cancer immunotherapy, and anti-tumor immunity via CAR-T-cells has been summarized elsewhere [35].

Similar to APCs, gene delivery to T-Cells is technically challenging [36]; current *ex vivo* approaches rely on isolating T-cells, genetically modifying them, selectively expanding positive clones and infusing the modified cells back into the patient. This is not only an expensive process but also requires sophisticated infrastructure and expertise. However, in a recent study, gene delivery via rationally designed multifunctional polymeric nanoparticles was introduced as an accessible, stable, and cost-effective alternative. These polymeric nanoparticles complexed with pDNA were designed to be taken up by T cells after in vivo administration and to localize to the nucleus after uptake. Tumor regression in a mouse model was as efficient using this *in vivo* approach as with conventional *ex vivo* methods [37].

Immunoengineering is not restricted to delivering DNA or mRNA, but can also be siRNA-mediated. Delivery of siRNA can lead to inhibition of immunosuppressive signaling molecules by downregulation or knock-down of the corresponding genes [28c], suggesting an alternative therapeutic approach for viral diseases such as Hepatitis B [38]. Currently approved siRNA therapies are in large for the treatment of rare genetic disorders as outline in [39].

2.4 Genome engineering

Recent advances in molecular biology field have led to the emergence of platform genome editing technologies, such as “zinc-finger nucleases” (ZFN), “transcription activator-like effector nuclease” (TALEN) [40], “clustered regularly spaced palindromic repeat” (CRISPR) with “CRISPR associated protein 9” (Cas9) [41], and, most recently, “retron library recombineering” (RLR) [42]. All of the mentioned tools enabled precise, site specific alteration in genome, which can be exploited for correction of genetic disorders. Since changes/modifications are permanently introduced into the host genome they are inherited to next generation of cells upon cell division, i.e., stably propagated and can therefore serve in potential therapeutic approach for treatment of both inherited genetic disorders such as Hemophilia, as well as acquired genetic disorders, e.g. cancer. Moreover, by *in vivo* administration of CRISPR/Cas9 as well as other genome editing tools, different types of permanent mutations could be introduced to generate animal disease models as research tools. [42].

CRISPR/Cas9, originated from an intricate bacterial immune system [43], has gained significant attention in recent years compared with ZFs and TALEs because of its high sequence-targeting fidelity, editing efficiency, and ease of use. CRISPR editing process as used in genetic engineering consists of two major components mostly supplied to cells via transfection; Cas9 (encoded by a nucleic acid or directly as protein) and single-guide RNA (sgRNA). Cas9 is an endonuclease enzyme capable of cleaving

double-strand DNA. Only when associated with a guide RNA, it constitutes an active Cas9-gRNA ribonucleoprotein (RNP). Upon formation of complementary base pairing between sgRNA and target sequence in genome, a double strand breakage (DSB) is generated by Cas9. Subsequently, by harnessing endogenous cellular repair mechanisms, either knock-in, insertion of an exogenous genes, or knock-out, deletion of existing gene can be achieved. Two main DNA repair mechanisms involved are non-homologous end joining (NHEJ), and homology directed repair (HDR). NHEJ occurs after cutting the chromosome by Cas9, resulting sometimes in the **in**sertion of a few nucleotides, but more often in short **de**letions (indel), both likely to result in the inactivation of the targeted gene, i.e., creating a knock-out. However, when an exogenous DNA fragment containing extra homology regions identical to chromosomal sequences at the site of Cas9 cutting is provided along with the RNP, this DNA sequence will be inserted between the broken arms of genomic DNA by a homologous recombination process [44].

Despite outperforming other genome editing technologies, CRISPR application is impeded in many cases due to delivery issue. Cas9 nucleases are large proteins (mostly >100kDa in size, depending on the species of origin), which are not easily transported through hydrophobic cell membranes. In fact, this protein has been co-transfected along with gRNA and also other DNA segments Alternatives to transport of protein molecules are transfection of either pDNA or mRNA coding for Cas9, which could be co-delivered simultaneously with sgRNA, or preferably before sgRNA in another step [45]. Different types of carrier systems have been designed for *in vitro* as well as *in vivo* delivery of either of the three above mentioned cargos for CRSPR/Cas9 genome editing, which is summarized comprehensively in other reviews; see ref. [46].

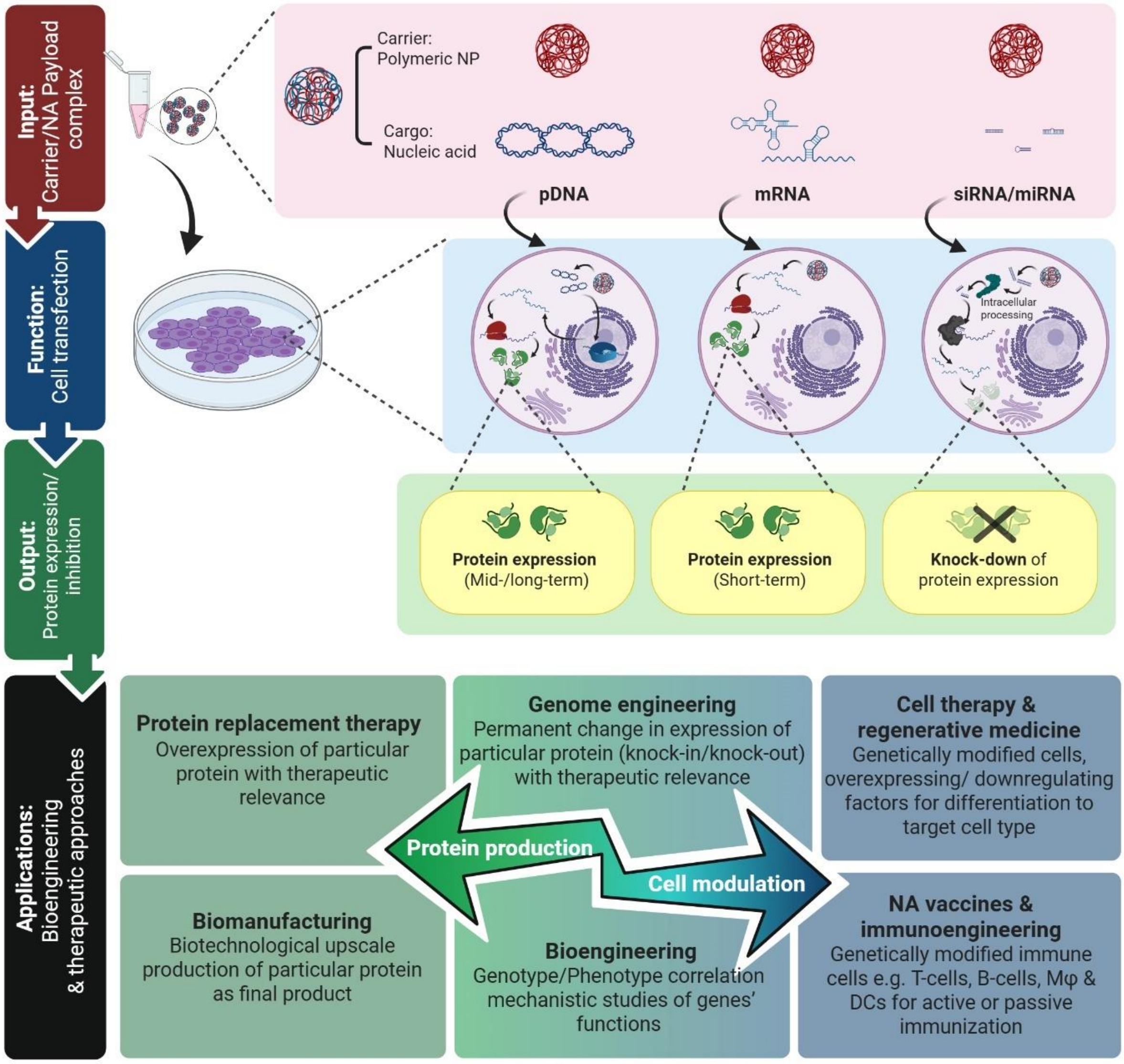

Fig. S1 Schematic representation of gene carrier systems in terms of input, function, and output categorized based on various types of cargos, i.e. pDNA, mRNA and siRNA/miRNA (Top panel); Overview of wide ranges of applications for gene delivery systems (Lower panel). Intracellular pathways of pDNA, mRNA and siRNA are highly schematized.

Table S1. Structural properties, delivery requirements, and therapeutic relevance of nucleic acid payloads

Payload	Size	Intracellular site of action	Key barriers	Structural features	Delivery requirements	Therapeutic relevance/examples	References
**Plasmid DNA (pDNA)**	Several kb; >3,000 nt; >2 MDa	Nucleus	Nuclear envelope, nucleases	Circular, double-stranded, with replication origin, promoter, selection markers	Must reach nucleus for transcription; protected from degradation	Expression vectors; gene therapy	[47]
**mRNA**	500–10,000 nt (~0.3–3 MDa)	Cytoplasm	Nucleases, instability, innate immune recognition	Single-stranded; IVT modifications (5′ cap, poly(A), UTRs, modified bases)	Cytoplasmic stability, reduced immunogenicity, efficient translation	Vaccines (e.g., SARS-CoV-2), protein replacement	[22, 48]
**Self-amplifying RNA (saRNA)**	>7 kb; ~3–4 MDa	Cytoplasm	Large size, innate immune recognition	Derived from alphaviruses; encodes nsP1–4 replicase + GOI	Cytoplasmic delivery at low doses; requires replication machinery	Prolonged expression; vaccines (influenza, Ebola, SARS-CoV-2)	[49]
**Small RNAs (siRNA, miRNA, shRNA)**	~21–23 nt (~7 kDa)	Cytoplasm (for mRNA cleavage)	Nuclease degradation, endosomal escape	Short duplexes or precursors processed by Dicer/RISC	Cytoplasmic delivery; stable duplex formation	RNAi therapies; gene silencing	[50]
**Ribozymes**	30–150 nt (~10–50 kDa)	Cytoplasm or nucleus (depending on target RNA)	Nuclease degradation, delivery efficiency	Catalytic RNA with self-cleaving or splicing activity	Requires stabilization and efficient cytosolic/nuclear delivery	Investigated for muscular dystrophy and viral targets	[51]
**CRISPR sgRNA**	~100 nt (~30 kDa)	Nucleus (with Cas nuclease)	Stability, nuclear import, off-target effects	Guide RNA forming Cas9 RNP complex	Delivery depends on format (pDNA, mRNA, or RNP)	Genome editing	[52]
**Other oligonucleotides (ASOs, aptamers, ssDNA)**	15–25 nt (~5–8 kDa)	Nucleus (ASOs); cytoplasm (aptamers)	Nuclease degradation, nuclear transport	ssDNA/RNA; aptamers fold into 3D binding structures	Stabilization by chemical modifications; nuclear import for ASOs	FDA-approved drugs: Nusinersen, Eteplirsen; Pegaptanib	[53]
**Diagnostic probes (beacons, FRET probes)**	15–30 nt	Cytoplasm	Nuclease degradation, background signal	Engineered oligonucleotides with reporter/quencher	Intracellular stability and signal specificity	Imaging and diagnostics (not therapeutic)	[54]

Table S2. Structural and functional properties of commonly used cationic polymers for nucleic acid delivery

Polymer	Structure / Key Features	Advantages	Limitations	Representative Applications & Studies
**Poly(L-lysine) (PLL)**	Linear poly(amino acid); α-PLL or ε-PLL with dense primary amines [55]	Biocompatible, low immunogenicity, efficient NA condensation	Poor buffering capacity, limited endosomal escape; requires additives (e.g., chloroquine, glycerol) [56]	Early gene transfer studies [57]; PEG-block copolymers improve morphology and transfection [58]
**Poly(amidoamine) (PAMAM)**	Highly branched dendrimer with defined generations; surface primary amines, internal tertiary amines [59]	Efficient NA condensation, buffering for endosomal escape, tunable size via generations, functionalizable surface	Complex synthesis, potential toxicity at high dose	Efficient siRNA/pDNA delivery without escape enhancers [60]; DNA/RNA nanocubes with PAMAM show delivery & immune effects [61]
**Poly(ethylene imine) (PEI)**	Branched (bPEI) or linear (lPEI); high charge density, secondary/tertiary amines [62]	"Gold standard" polymer; strong condensation; efficient endosomal escape via proton sponge/membrane interaction; versatile modifications [63]	High cytotoxicity, preparation-dependent transfection outcomes	Benchmark for transfection [64]; split-Ugi library for immune cell targeting [65]; saRNA vaccines with compact polyplexes [66]
**Poly(dimethylaminoethyl methacrylate) (PDMAEMA)**	Tertiary amine-containing acrylic polymer; tunable MW and pKa; thermo- and pH-sensitive [67]	Easily incorporated into copolymers via RDRP; low cytotoxicity; tunable architectures (graft, block, star) [68]	Moderate efficiency compared to PEI; requires optimization	mPEG-GO/PDMAEMA nanohybrids for siRNA delivery with photothermal potential [69]
**Poly(β-amino esters) (PBAEs)**	Degradable cationic polymers with ester linkages; synthesized via aza-Michael addition [70]	Biodegradable, modular chemistry, high versatility; reduced long-term toxicity	Stability and cytotoxicity concerns depending on composition	Library of 55 PBAEs identified sustained mRNA expression in vivo; amine 8 (5-amino-1-pentanol) polymers showed best uptake and expression [71]

Table S3. Key physicochemical parameters of polymeric carriers and their mechanistic impact on NA delivery

Parameter	Relevant design aspects	Mechanistic impact	Key trade-offs	Representative refs
**Charge density**	Monomer pKa, copolymer ratio	Condensation, stability, uptake, escape, unpacking	High charge ↑ transfection but causes cytotoxicity & poor unpacking	[72]
**Molecular weight (MW)**	Degree of polymerization, hydrolysis level	Condensation strength, particle size, expression	Higher MW ↑ efficiency but ↑ toxicity; payload-specific optima	[73]
**Hydrophobicity**	Side-chain length, hydrophobic block ratio	Membrane interaction, escape, stability, tissue tropism	Excess hydrophobicity → hemolysis & serum interactions	[74]
**Topology (architecture)**	Linear vs branched vs dendrimer, graft density	Charge distribution, condensation, escape, unpacking	Structure alters efficiency–toxicity balance	[75]
**Chain rigidity**	Backbone chemistry, side-chain bulk	Binding strength, stability, escape, bioavailability	Too rigid → weak complexation; too flexible → over-stabilization	[75b, 76]
**Surface charge (ζ)**	Terminal group chemistry, PEGylation	Uptake, escape, circulation stability	Positive charge aids uptake but ↑ aggregation	[77]
**Colloidal stability**	Block copolymer corona, MW of shielding segment	Protection, aggregation, reproducibility	Over-stability hinders unpacking	[78]
**Stimuli-responsiveness**	pH-sensitive linkers, disulfides, ROS-/ATP-cleavable bonds	Escape, release, targeting	Must match trigger & tissue; safety critical	[79]

Refrences

[1] F. Crick,Central dogma of molecular biology, *Nature* **1970**, *227* (5258), 561, https://doi.org/10.1038/227561a0.

[2] a) J. A. Wolff, J. Lederberg,An early history of gene transfer and therapy, *Human gene therapy* **1994**, *5* (4), 469, https://doi.org/10.1089/hum.1994.5.4-469; b) T. Friedmann,A brief history of gene therapy, *Nature genetics* **1992**, *2* (2), 93, https://doi.org/10.1038/ng1092-93.

[3] a) C. E. Thomas, A. Ehrhardt, M. A. Kay,Progress and problems with the use of viral vectors for gene therapy, *Nature reviews. Genetics* **2003**, *4* (5), 346, https://doi.org/10.1038/nrg1066; b) L. Vannucci, M. Lai, F. Chiuppesi, L. Ceccherini-Nelli, M. Pistello,Viral vectors: a look back and ahead on gene transfer technology, *The new microbiologica* **2013**, *36* (1), 1; c) R. Waehler, S. J. Russell, D. T. Curiel,Engineering targeted viral vectors for gene therapy, *Nature Reviews Genetics* **2007**, *8* (8), 573, https://doi.org/10.1038/nrg2141.

[4] a) D. J. Wells,Gene Therapy Progress and Prospects: Electroporation and other physical methods, *Gene therapy* **2004**, *11* (18), 1363, https://doi.org/10.1038/sj.gt.3302337; b) S. Li,Electroporation gene therapy: new developments in vivo and in vitro, *Current gene therapy* **2004**, *4* (3), 309, https://doi.org/10.2174/1566523043346336; c) L. M. Mir,Application of electroporation gene therapy: past, current, and future, *Methods in molecular biology (Clifton, N.J.)* **2008**, *423*, 3, https://doi.org/10.1007/978-1-59745-194-9_1.

[5] a) C. M. H. Newman, T. Bettinger,Gene therapy progress and prospects: Ultrasound for gene transfer, *Gene therapy* **2007**, *14* (6), 465, https://doi.org/10.1038/sj.gt.3302925; b) M. Wang, Y. Zhang, C. Cai, J. Tu, X. Guo, D. Zhang,Sonoporation-induced cell membrane permeabilization and cytoskeleton disassembly at varied acoustic and microbubble-cell parameters, *Scientific reports* **2018**, *8* (1), 3885, https://doi.org/10.1038/s41598-018-22056-8.

[6] P. Chakrabarty, P. Gupta, K. Illath, S. Kar, M. Nagai, F. G. Tseng, T. S. Santra,Microfluidic mechanoporation for cellular delivery and analysis, *Mater Today Bio* **2022**, *13*, 100193, https://doi.org/10.1016/j.mtbio.2021.100193.

[7] N.-S. Yang, W. H. Sun,Gene gun and other non-viral approaches for cancer gene therapy, *Nature Medicine* **1995**, *1* (5), 481, https://doi.org/10.1038/nm0595-481.

[8] a) Y. T. Chow, S. Chen, R. Wang, C. Liu, C.-w. Kong, R. A. Li, S. H. Cheng, D. Sun,Single Cell Transfection through Precise Microinjection with Quantitatively Controlled Injection Volumes, *Scientific reports* **2016**, *6* (1), 24127, https://doi.org/10.1038/srep24127; b) W. Xu,Microinjection and Micromanipulation: A Historical Perspective, *Methods in molecular biology (Clifton, N.J.)* **2019**, *1874*, 1, https://doi.org/10.1007/978-1-4939-8831-0_1; c) V. A. S. Jones, M. Bucher, E. A. Hambleton, A. Guse,Microinjection to deliver protein, mRNA, and DNA into zygotes of the cnidarian endosymbiosis model Aiptasia sp, *Scientific reports* **2018**, *8* (1), 16437, https://doi.org/10.1038/s41598-018-34773-1.

[9] H. Yin, R. L. Kanasty, A. A. Eltoukhy, A. J. Vegas, J. R. Dorkin, D. G. Anderson,Non-viral vectors for gene-based therapy, *Nature Reviews Genetics* **2014**, *15* (8), 541, https://doi.org/10.1038/nrg3763.

[10] W. Weiwei, D. Zijun, X. Xun, L. Zhengdong, J. Friedrich, Nan Ma, L. Andreas,Functional Nanoparticles and their Interactions with Mesenchymal Stem Cells, *Current pharmaceutical design* **2017**, *23* (26), 3814, https://doi.org/http://dx.doi.org/10.2174/1381612823666170622110654.

[11] a) C. Zylberberg, K. Gaskill, S. Pasley, S. Matosevic,Engineering liposomal nanoparticles for targeted gene therapy, *Gene therapy* **2017**, *24* (8), 441, https://doi.org/10.1038/gt.2017.41; b) J. A. Kulkarni, P. R. Cullis, R. van der Meel,Lipid Nanoparticles Enabling Gene Therapies: From Concepts to Clinical Utility, *Nucleic acid therapeutics* **2018**, *28* (3), 146, https://doi.org/10.1089/nat.2018.0721; c) A. A. Barba, S. Bochicchio, A. Dalmoro, G. Lamberti,Lipid Delivery Systems for Nucleic-Acid-Based-Drugs: From Production to Clinical Applications, *Pharmaceutics* **2019**, *11* (8), https://doi.org/10.3390/pharmaceutics11080360.

[12] M. Kwon, B. L. Firestein,DNA transfection: calcium phosphate method, *Methods in molecular biology (Clifton, N.J.)* **2013**, *1018*, 107, https://doi.org/10.1007/978-1-62703-444-9_10.

[13] Y. K. Ho, J. Y. Woo, G. X. E. Tu, L. W. Deng, H. P. Too,A highly efficient non-viral process for programming mesenchymal stem cells for gene directed enzyme prodrug cancer therapy, *Scientific reports* **2020**, *10* (1), 14257, https://doi.org/10.1038/s41598-020-71224-2.

[14] X. Zhou, R. Hao, C. Chen, Z. Su, L. Zhao, Z. Luo, W. Xie,Rapid Delivery of Nanobodies/V(H)Hs into Living Cells via Expressing In Vitro-Transcribed mRNA, *Molecular therapy. Methods & clinical development* **2020**, *17*, 401, https://doi.org/10.1016/j.omtm.2020.01.008.

[15] A. Lang, J. Neuhaus, M. Pfeiffenberger, E. Schröder, I. Ponomarev, Y. Weber, T. Gaber, M. F. Schmidt,Optimization of a nonviral transfection system to evaluate Cox-2 controlled interleukin-4 expression for osteoarthritis gene therapy in vitro, *The journal of gene medicine* **2014**, *16* (11-12), 352, https://doi.org/10.1002/jgm.2812.

[16] a) P. S. Kowalski, A. Rudra, L. Miao, D. G. Anderson,Delivering the Messenger: Advances in Technologies for Therapeutic mRNA Delivery, *Molecular therapy : the journal of the American Society of Gene Therapy* **2019**, *27* (4), 710, https://doi.org/10.1016/j.ymthe.2019.02.012; b) E. Tambuyzer, B. Vandendriessche, C. P. Austin, P. J. Brooks, K. Larsson, K. I. Miller Needleman, J. Valentine, K. Davies, S. C. Groft, R. Preti, T. I. Oprea, M. Prunotto,Therapies for rare diseases: therapeutic modalities, progress and challenges ahead, *Nature Reviews Drug Discovery* **2020**, *19* (2), 93, https://doi.org/10.1038/s41573-019-0049-9.

[17] J. Zhao, H. T. Yang, L. Wasala, K. Zhang, Y. Yue, D. Duan, Y. Lai,Dystrophin R16/17 protein therapy restores sarcolemmal nNOS in trans and improves muscle perfusion and function, *Molecular Medicine* **2019**, *25* (1), 31, https://doi.org/10.1186/s10020-019-0101-6.

[18] a) S. Ramaswamy, N. Tonnu, K. Tachikawa, P. Limphong, J. B. Vega, P. P. Karmali, P. Chivukula, I. M. Verma,Systemic delivery of factor IX messenger RNA for protein replacement therapy, *Proceedings of the National Academy of Sciences of the United States of America* **2017**, *114* (10), E1941, https://doi.org/10.1073/pnas.1619653114; b) C. J. Stephens, E. Kashentseva, W. Everett, L. Kaliberova, D. T. Curiel,Targeted in vivo knock-in of human alpha-1-antitrypsin cDNA using adenoviral delivery of CRISPR/Cas9, *Gene therapy* **2018**, *25* (2), 139, https://doi.org/10.1038/s41434-018-0003-1; c) L. Deng, X. Gao, G. Fan, C. Yang,Effects of GDNF-Transfected Marrow Stromal Cells on Rats with Intracerebral Hemorrhage, *Journal of stroke and cerebrovascular diseases : the official journal of National Stroke Association* **2019**, *28* (9), 2555, https://doi.org/10.1016/j.jstrokecerebrovasdis.2019.06.002.

[19] L. Baldi, D. L. Hacker, M. Adam, F. M. Wurm,Recombinant protein production by large-scale transient gene expression in mammalian cells: state of the art and future perspectives, *Biotechnology letters* **2007**, *29* (5), 677, https://doi.org/10.1007/s10529-006-9297-y.

[20] Z. Li, I. P. Michael, D. Zhou, A. Nagy, J. M. Rini,Simple piggyBac transposon-based mammalian cell expression system for inducible protein production, *Proceedings of the National Academy of Sciences of the United States of America* **2013**, *110* (13), 5004, https://doi.org/10.1073/pnas.1218620110.

[21] W. Li, N. Ma, L.-L. Ong, C. Nesselmann, C. Klopsch, Y. Ladilov, D. Furlani, C. Piechaczek, J. M. Moebius, K. Lützow, A. Lendlein, C. Stamm, R.-K. Li, G. Steinhoff,Bcl-2 Engineered MSCs Inhibited Apoptosis and Improved Heart Function, *STEM CELLS* **2007**, *25* (8), 2118, https://doi.org/https://doi.org/10.1634/stemcells.2006-0771.

[22] H. Youn, J. K. Chung,Modified mRNA as an alternative to plasmid DNA (pDNA) for transcript replacement and vaccination therapy, *Expert opinion on biological therapy* **2015**, *15* (9), 1337, https://doi.org/10.1517/14712598.2015.1057563.

[23] Y. Xiao, Y. Jiang, H. Song, T. Liang, Y. Li, D. Yan, Q. Fu, Z. Li,RNF7 knockdown inhibits prostate cancer tumorigenesis by inactivation of ERK1/2 pathway, *Scientific reports* **2017**, *7* (1), 43683, https://doi.org/10.1038/srep43683.

[24] a) G. Zhao, J. Cui, J. G. Zhang, Q. Qin, Q. Chen, T. Yin, S. C. Deng, Y. Liu, L. Liu, B. Wang, K. Tian, G. B. Wang, C. Y. Wang,SIRT1 RNAi knockdown induces apoptosis and senescence, inhibits invasion and enhances chemosensitivity in pancreatic cancer cells, *Gene therapy* **2011**, *18* (9), 920, https://doi.org/10.1038/gt.2011.81; b) R. S. Soderquist, L. Crawford, E. Liu, M. Lu, A. Agarwal, G. R. Anderson, K. H. Lin, P. S. Winter, M. Cakir, K. C. Wood,Systematic mapping of BCL-2 gene dependencies in cancer reveals molecular determinants of BH3 mimetic sensitivity, *Nature Communications* **2018**, *9*

(1), 3513, https://doi.org/10.1038/s41467-018-05815-z; c) L. Wei, W. Qu, J. Sun, X. Wang, L. Lv, L. Xie, X. Song,Knockdown of cancerous inhibitor of protein phosphatase 2A may sensitize NSCLC cells to cisplatin, *Cancer Gene Therapy* **2014**, *21* (5), 194, https://doi.org/10.1038/cgt.2014.18.
[25] L. A. L. Fliervoet, H. Zhang, E. van Groesen, K. Fortuin, N. Duin, K. Remaut, R. M. Schiffelers, W. E. Hennink, T. Vermonden,Local release of siRNA using polyplex-loaded thermosensitive hydrogels, *Nanoscale* **2020**, *12* (18), 10347, https://doi.org/10.1039/d0nr03147j.
[26] a) R. Kanasty, J. R. Dorkin, A. Vegas, D. Anderson,Delivery materials for siRNA therapeutics, *Nature Materials* **2013**, *12* (11), 967, https://doi.org/10.1038/nmat3765; b) J. O. Eloy, R. Petrilli, R. F. Lopez, R. J. Lee,Stimuli-Responsive Nanoparticles for siRNA Delivery, *Current pharmaceutical design* **2015**, *21* (29), 4131, https://doi.org/10.2174/1381612821666150901095349; c) K. A. Whitehead, R. Langer, D. G. Anderson,Knocking down barriers: advances in siRNA delivery, *Nature Reviews Drug Discovery* **2009**, *8* (2), 129, https://doi.org/10.1038/nrd2742.
[27] M. Nestor, B. Berman, P. Lu, M. Molyneaux,Safety and Efficacy of TGF-β1/COX-2 Silencing Therapeutic in Adults With Cutaneous Squamous Cell Carcinoma In Situ, *J Drugs Dermatol* **2022**, *21* (5), 472, https://doi.org/10.36849/jdd.6384.
[28] a) N. P. Restifo, H. Ying, L. Hwang, W. W. Leitner,The promise of nucleic acid vaccines, *Gene therapy* **2000**, *7* (2), 89, https://doi.org/10.1038/sj.gt.3301117; b) N. Pardi, M. J. Hogan, F. W. Porter, D. Weissman,mRNA vaccines - a new era in vaccinology, *Nature reviews. Drug discovery* **2018**, *17* (4), 261, https://doi.org/10.1038/nrd.2017.243; c) S. Y. Tzeng, J. J. Green,Polymeric Nucleic Acid Delivery for Immunoengineering, *Current opinion in biomedical engineering* **2018**, *7*, 42, https://doi.org/10.1016/j.cobme.2018.09.005.
[29] J. Lai, S. Mardiana, I. G. House, K. Sek, M. A. Henderson, L. Giuffrida, A. X. Y. Chen, K. L. Todd, E. V. Petley, J. D. Chan, E. M. Carrington, A. M. Lew, B. J. Solomon, J. A. Trapani, K. Kedzierska, M. Evrard, S. J. Vervoort, J. Waithman, P. K. Darcy, P. A. Beavis,Adoptive cellular therapy with T cells expressing the dendritic cell growth factor Flt3L drives epitope spreading and antitumor immunity, *Nature Immunology* **2020**, *21* (8), 914, https://doi.org/10.1038/s41590-020-0676-7.
[30] a) H. F. Florindo, R. Kleiner, D. Vaskovich-Koubi, R. C. Acúrcio, B. Carreira, E. Yeini, G. Tiram, Y. Liubomirski, R. Satchi-Fainaro,Immune-mediated approaches against COVID-19, *Nature nanotechnology* **2020**, https://doi.org/10.1038/s41565-020-0732-3; b) COVID-19 therapies and vaccine landscape, *Nature Materials* **2020**, *19* (8), 809, https://doi.org/10.1038/s41563-020-0758-9.
[31] a) J. Rice, C. H. Ottensmeier, F. K. Stevenson,DNA vaccines: precision tools for activating effective immunity against cancer, *Nature Reviews Cancer* **2008**, *8* (2), 108, https://doi.org/10.1038/nrc2326; b) M. A. Kutzler, D. B. Weiner,DNA vaccines: ready for prime time?, *Nature Reviews Genetics* **2008**, *9* (10), 776, https://doi.org/10.1038/nrg2432.
[32] N. Pardi, M. J. Hogan, F. W. Porter, D. Weissman,mRNA vaccines — a new era in vaccinology, *Nature Reviews Drug Discovery* **2018**, *17* (4), 261, https://doi.org/10.1038/nrd.2017.243.
[33] W. Ho, M. Gao, F. Li, Z. Li, X. Q. Zhang, X. Xu,Next-Generation Vaccines: Nanoparticle-Mediated DNA and mRNA Delivery, *Advanced healthcare materials* **2021**, e2001812, https://doi.org/10.1002/adhm.202001812.
[34] J. D. Beck, D. Reidenbach, N. Salomon, U. Sahin, Ö. Türeci, M. Vormehr, L. M. Kranz,mRNA therapeutics in cancer immunotherapy, *Mol Cancer* **2021**, *20* (1), 69, https://doi.org/10.1186/s12943-021-01348-0.
[35] a) K. N. Dzhandzhugazyan, P. Guldberg, A. F. Kirkin,Adoptive T cell cancer therapy, *Nature Materials* **2018**, *17* (6), 475, https://doi.org/10.1038/s41563-018-0094-5; b) D. Li, X. Li, W.-L. Zhou, Y. Huang, X. Liang, L. Jiang, X. Yang, J. Sun, Z. Li, W.-D. Han, W. Wang,Genetically engineered T cells for cancer immunotherapy, *Signal Transduction and Targeted Therapy* **2019**, *4* (1), 35, https://doi.org/10.1038/s41392-019-0070-9.
[36] D. Delcassian, S. Sattler, I. E. Dunlop,T cell immunoengineering with advanced biomaterials, *Integrative biology : quantitative biosciences from nano to macro* **2017**, *9* (3), 211, https://doi.org/10.1039/c6ib00233a.

[37] T. T. Smith, S. B. Stephan, H. F. Moffett, L. E. McKnight, W. Ji, D. Reiman, E. Bonagofski, M. E. Wohlfahrt, S. P. S. Pillai, M. T. Stephan,In situ programming of leukaemia-specific T cells using synthetic DNA nanocarriers, *Nature nanotechnology* **2017**, *12* (8), 813, https://doi.org/10.1038/nnano.2017.57.
[38] F. van den Berg, S. W. Limani, N. Mnyandu, M. B. Maepa, A. Ely, P. Arbuthnot,Advances with RNAi-Based Therapy for Hepatitis B Virus Infection, *Viruses* **2020**, *12* (8), https://doi.org/10.3390/v12080851.
[39] L. Capelôa, M. Yazdi, H. Zhang, X. Chen, Y. Nie, E. Wagner, U. Lächelt, M. Barz,Cross-Linkable Polyion Complex Micelles from Polypept(o)ide-Based ABC-Triblock Copolymers for siRNA Delivery, *Macromol Rapid Commun* **2022**, *43* (12), e2100698, https://doi.org/10.1002/marc.202100698.
[40] H. Li, Y. Yang, W. Hong, M. Huang, M. Wu, X. Zhao,Applications of genome editing technology in the targeted therapy of human diseases: mechanisms, advances and prospects, *Signal Transduction and Targeted Therapy* **2020**, *5* (1), 1, https://doi.org/10.1038/s41392-019-0089-y.
[41] A. V. Anzalone, L. W. Koblan, D. R. Liu,Genome editing with CRISPR–Cas nucleases, base editors, transposases and prime editors, *Nature biotechnology* **2020**, *38* (7), 824, https://doi.org/10.1038/s41587-020-0561-9.
[42] M. G. Schubert, D. B. Goodman, T. M. Wannier, D. Kaur, F. Farzadfard, T. K. Lu, S. L. Shipman, G. M. Church,High-throughput functional variant screens via in vivo production of single-stranded DNA, *Proceedings of the National Academy of Sciences of the United States of America* **2021**, *118* (18), https://doi.org/10.1073/pnas.2018181118.
[43] a) J. A. Doudna, E. Charpentier,Genome editing. The new frontier of genome engineering with CRISPR-Cas9, *Science* **2014**, *346* (6213), 1258096, https://doi.org/10.1126/science.1258096; b) F. Hille, H. Richter, S. P. Wong, M. Bratovič, S. Ressel, E. Charpentier,The Biology of CRISPR-Cas: Backward and Forward, *Cell* **2018**, *172* (6), 1239, https://doi.org/10.1016/j.cell.2017.11.032.
[44] a) M. Adli,The CRISPR tool kit for genome editing and beyond, *Nature Communications* **2018**, *9* (1), 1911, https://doi.org/10.1038/s41467-018-04252-2; b) S. B. Moon, D. Y. Kim, J.-H. Ko, Y.-S. Kim,Recent advances in the CRISPR genome editing tool set, *Experimental & Molecular Medicine* **2019**, *51* (11), 1, https://doi.org/10.1038/s12276-019-0339-7.
[45] a) M. Mehravar, A. Shirazi, M. M. Mehrazar, M. Nazari, M. Banan,CRISPR/Cas9 System for Efficient Genome Editing and Targeting in the Mouse NIH/3T3 Cells, *Avicenna journal of medical biotechnology* **2019**, *11* (2), 149; b) H. X. Zhang, Y. Zhang, H. Yin,Genome Editing with mRNA Encoding ZFN, TALEN, and Cas9, *Molecular therapy : the journal of the American Society of Gene Therapy* **2019**, *27* (4), 735, https://doi.org/10.1016/j.ymthe.2019.01.014.
[46] a) J. Eoh, L. Gu,Biomaterials as vectors for the delivery of CRISPR-Cas9, *Biomaterials science* **2019**, *7* (4), 1240, https://doi.org/10.1039/c8bm01310a; b) Z. Glass, M. Lee, Y. Li, Q. Xu,Engineering the Delivery System for CRISPR-Based Genome Editing, *Trends in biotechnology* **2018**, *36* (2), 173, https://doi.org/10.1016/j.tibtech.2017.11.006; c) D. C. Luther, Y. W. Lee, H. Nagaraj, F. Scaletti, V. M. Rotello,Delivery approaches for CRISPR/Cas9 therapeutics in vivo: advances and challenges, *Expert opinion on drug delivery* **2018**, *15* (9), 905, https://doi.org/10.1080/17425247.2018.1517746.
[47] A. K. Pannier, L. D. Shea,Controlled release systems for DNA delivery, *Molecular Therapy* **2004**, *10* (1), 19, https://doi.org/https://doi.org/10.1016/j.ymthe.2004.03.020.
[48] a) Z. Meng, J. O'Keeffe-Ahern, J. Lyu, L. Pierucci, D. Zhou, W. Wang,A new developing class of gene delivery: messenger RNA-based therapeutics, *Biomaterials science* **2017**, *5* (12), 2381, https://doi.org/10.1039/c7bm00712d; b) U. Sahin, K. Karikó, Ö. Türeci,mRNA-based therapeutics--developing a new class of drugs, *Nature reviews. Drug discovery* **2014**, *13* (10), 759, https://doi.org/10.1038/nrd4278; c) J. Devoldere, H. Dewitte, S. C. De Smedt, K. Remaut,Evading innate immunity in nonviral mRNA delivery: don't shoot the messenger, *Drug discovery today* **2016**, *21* (1), 11, https://doi.org/10.1016/j.drudis.2015.07.009; d) E. M. Harcourt, A. M. Kietrys, E. T. Kool,Chemical and structural effects of base modifications in messenger RNA, *Nature* **2017**, *541* (7637), 339, https://doi.org/10.1038/nature21351; e) S. H. Boo, Y. K. Kim,The emerging role of RNA modifications in the regulation of mRNA stability, *Exp Mol Med* **2020**, *52* (3), 400, https://doi.org/10.1038/s12276-020-0407-z; f) D. M. Mauger, B. J. Cabral, V. Presnyak, S. V. Su, D. W. Reid, B. Goodman, K. Link, N. Khatwani, J. Reynders, M. J. Moore, I. J. McFadyen,mRNA structure

regulates protein expression through changes in functional half-life, *Proceedings of the National Academy of Sciences of the United States of America* **2019**, *116* (48), 24075, https://doi.org/10.1073/pnas.1908052116.
[49] a) K. Bloom, F. van den Berg, P. Arbuthnot,Self-amplifying RNA vaccines for infectious diseases, *Gene therapy* **2021**, *28* (3), 117, https://doi.org/10.1038/s41434-020-00204-y; b) T. Beissert, L. Koste, M. Perkovic, K. C. Walzer, S. Erbar, A. Selmi, M. Diken, S. Kreiter, Ö. Türeci, U. Sahin,Improvement of In Vivo Expression of Genes Delivered by Self-Amplifying RNA Using Vaccinia Virus Immune Evasion Proteins, *Human gene therapy* **2017**, *28* (12), 1138, https://doi.org/10.1089/hum.2017.121.
[50] a) R. W. Carthew, E. J. Sontheimer,Origins and Mechanisms of miRNAs and siRNAs, *Cell* **2009**, *136* (4), 642, https://doi.org/10.1016/j.cell.2009.01.035; b) D. Zhou, Q. S. He, C. Wang, J. Zhang, F. Wong-Staal,RNA interference and potential applications, *Current topics in medicinal chemistry* **2006**, *6* (9), 901, https://doi.org/10.2174/156802606777303630; c) J. K. Lam, M. Y. Chow, Y. Zhang, S. W. Leung,siRNA Versus miRNA as Therapeutics for Gene Silencing, *Molecular therapy. Nucleic acids* **2015**, *4* (9), e252, https://doi.org/10.1038/mtna.2015.23.
[51] a) W. G. Scott,Ribozymes, *Current Opinion in Structural Biology* **2007**, *17* (3), 280, https://doi.org/https://doi.org/10.1016/j.sbi.2007.05.003; b) S. R. Lindley, K. C. V. Subbaiah, F. Priyanka, P. Poosala, Y. Ma, L. Jalinous, J. A. West, W. A. Richardson, T. N. Thomas, D. M. Anderson,Ribozyme-activated mRNA trans-ligation enables large gene delivery to treat muscular dystrophies, *Science* **2024**, *386* (6723), 762, https://doi.org/doi:10.1126/science.adp8179.
[52] S. K. Alsaiari, B. Eshaghi, B. Du, M. Kanelli, G. Li, X. Wu, L. Zhang, M. Chaddah, A. Lau, X. Yang, R. Langer, A. Jaklenec,CRISPR–Cas9 delivery strategies for the modulation of immune and non-immune cells, *Nature Reviews Materials* **2025**, *10* (1), 44, https://doi.org/10.1038/s41578-024-00725-7.
[53] a) D. Park, S. J. Lee, J.-W. Park,Aptamer-Based Smart Targeting and Spatial Trigger–Response Drug-Delivery Systems for Anticancer Therapy, *Biomedicines* **2024**, *12* (1), 187; b) M. Safarkhani, S. Ahmadi, H. Ipakchi, M. R. Saeb, P. Makvandi, M. Ebrahimi Warkiani, N. Rabiee, Y. Huh,Advancements in Aptamer-Driven DNA Nanostructures for Precision Drug Delivery, *Advanced Science* **2024**, *11* (26), 2401617, https://doi.org/https://doi.org/10.1002/advs.202401617; c) H. Mintz-Hittner,Intravitreal pegaptanib as adjunctive treatment for stage 3+ ROP shown to be effective in a prospective, randomized, controlled multicenter clinical trial, *European Journal of Ophthalmology* **2012**, *22* (5), 685, https://doi.org/10.5301/ejo.5000176; d) R. Günther, C. Wurster, S. Brakemeier, A. Osmanovic, O. Schreiber-Katz, S. Petri, Z. Uzelac, M. Hiebeler, S. Thiele, M. Walter, M. Weiler, T. Kessler, M. Freigang, H. Lapp, I. Cordts, P. Lingor, M. Deschauer, A. Hahn, K. Martakis, R. Steinbach, B. Ilse, A. Rödiger, J. Bellut, J. Nentwich, D. Zeller, M. Muhandes, T. Baum, J. Koch, B. Schrank, S. Fischer, A. Hermann, C. Kamm, S. Naegel, A. Mensch, M. Weber, C. Neuwirth, H. Lehmann, G. Wunderlich, C. Stadler, M. Tomforde, A. George, M. Gross, A. Pechmann, J. Kirschner, M. Türk, M. Schimmel, G. Bernert, P. Martin, C. Rauscher, G. Hörste, P. Baum, W. Löscher, M. Flotats-Bastardas, C. Köhler, K. Probst-Schendzielorz, S. Goldbach, U. Schara-Schmidt, W. Müller-Felber, H. Lochmüller, O. von Velsen, C. Kleinschnitz, A. Ludolph, T. Hagenacker, S. S. Grp,Long-term efficacy and safety of nusinersen in adults with 5q spinal muscular atrophy: a prospective European multinational observational study, *Lancet Regional Health-Europe* **2024**, *39*, https://doi.org/10.1016/j.lanepe.2024.100862; e) A. Aartsma-Rus, A. M. Krieg,FDA Approves Eteplirsen for Duchenne Muscular Dystrophy: The Next Chapter in the Eteplirsen Saga, *Nucleic acid therapeutics* **2016**, *27* (1), 1, https://doi.org/10.1089/nat.2016.0657.
[54] a) S. X. Han, X. Jia, J. L. Ma, Q. Zhu,Molecular beacons: a novel optical diagnostic tool, *Arch Immunol Ther Exp (Warsz)* **2013**, *61* (2), 139, https://doi.org/10.1007/s00005-012-0209-7; b) F. Peng, X. N. Ai, J. Sun, L. S. Yang, B. X. Gao,Recent advances in FRET probes for mitochondrial imaging and sensing, *CHEMICAL COMMUNICATIONS* **2024**, *60* (22), 2994, https://doi.org/10.1039/d4cc00018h.
[55] M. Männistö, S. Vanderkerken, V. Toncheva, M. Elomaa, M. Ruponen, E. Schacht, A. Urtti,Structure–activity relationships of poly(l-lysines): effects of pegylation and molecular shape on physicochemical and biological properties in gene delivery, *Journal of Controlled Release* **2002**, *83* (1), 169, https://doi.org/https://doi.org/10.1016/S0168-3659(02)00178-5.
[56] a) P. Erbacher, A. C. Roche, M. Monsigny, P. Midoux,Putative Role of Chloroquine in Gene Transfer into a Human Hepatoma Cell Line by DNA/Lactosylated Polylysine Complexes, *Experimental*

Cell Research **1996**, *225* (1), 186, https://doi.org/https://doi.org/10.1006/excr.1996.0169; b) W. Zauner, A. Kichler, W. Schmidt, A. Sinski, E. Wagner,Glycerol Enhancement of Ligand-Polylysine/DNA Transfection, *BioTechniques* **1996**, *20* (5), 905, https://doi.org/10.2144/96205rr04.
[57] F. E. Farber, J. L. Melnick, J. S. Butel,Optimal conditions for uptake of exogenous DNA by chinese hamster lung cells deficient in hypoxanthine-guanine phosphoribosyltransferase, *Biochimica et Biophysica Acta (BBA) - Nucleic Acids and Protein Synthesis* **1975**, *390* (3), 298, https://doi.org/https://doi.org/10.1016/0005-2787(75)90350-0.
[58] K. M. Takeda, K. Osada, T. A. Tockary, A. Dirisala, Q. Chen, K. Kataoka,Poly(ethylene glycol) Crowding as Critical Factor To Determine pDNA Packaging Scheme into Polyplex Micelles for Enhanced Gene Expression, *Biomacromolecules* **2017**, *18* (1), 36, https://doi.org/10.1021/acs.biomac.6b01247.
[59] D. A. Tomalia, H. Baker, J. Dewald, M. Hall, G. Kallos, S. Martin, J. Roeck, J. Ryder, P. Smith,A New Class of Polymers: Starburst-Dendritic Macromolecules, *Polymer Journal* **1985**, *17* (1), 117, https://doi.org/10.1295/polymj.17.117.
[60] J. Haensler, F. C. Szoka, Jr.,Polyamidoamine cascade polymers mediate efficient transfection of cells in culture, *Bioconjugate chemistry* **1993**, *4* (5), 372, https://doi.org/10.1021/bc00023a012.
[61] Y. I. Avila, L. P. Rebolledo, N. Leal Santos, B. Rawlins, Y. Radwan, M. Andrade-Muñoz, E. Skelly, M. R. Chandler, L. N. S. Andrade, T. J. Kim, M. A. Dobrovolskaia, K. A. Afonin,Changes in Generations of PAMAM Dendrimers and Compositions of Nucleic Acid Nanoparticles Govern Delivery and Immune Recognition, *ACS biomaterials science & engineering* **2025**, *11* (6), 3726, https://doi.org/10.1021/acsbiomaterials.5c00336.
[62] M. A. Mees, R. Hoogenboom,Full and partial hydrolysis of poly(2-oxazoline)s and the subsequent post-polymerization modification of the resulting polyethylenimine (co)polymers, *Polymer Chemistry* **2018**, *9* (40), 4968, https://doi.org/10.1039/C8PY00978C.
[63] a) T. Bus, A. Traeger, U. S. Schubert,The great escape: how cationic polyplexes overcome the endosomal barrier, *Journal of Materials Chemistry B* **2018**, *6* (43), 6904, https://doi.org/10.1039/C8TB00967H; b) J.-P. Behr,The Proton Sponge: a Trick to Enter Cells the Viruses Did Not Exploit, *CHIMIA* **1997**, *51* (1-2), 34, https://doi.org/10.2533/chimia.1997.34.
[64] O. Boussif, F. Lezoualc'h, M. A. Zanta, M. D. Mergny, D. Scherman, B. Demeneix, J. P. Behr,A versatile vector for gene and oligonucleotide transfer into cells in culture and in vivo: polyethylenimine, *Proceedings of the National Academy of Sciences* **1995**, *92* (16), 7297, https://doi.org/10.1073/pnas.92.16.7297.
[65] K. Y. Vlasova, A. Kerr, N. D. Pennock, A. Jozic, D. K. Sahel, M. Gautam, N. T. V. Murthy, A. Roberts, M. W. Ali, K. D. MacDonald, J. M. Walker, R. Luxenhofer, G. Sahay,Synthesis of ionizable lipopolymers using split-Ugi reaction for pulmonary delivery of various size RNAs and gene editing, *Nature Communications* **2025**, *16* (1), 4021, https://doi.org/10.1038/s41467-025-59136-z.
[66] J. Moreno Herrero, T. B. Stahl, S. Erbar, K. Maxeiner, A. Schlegel, T. Bacic, J. Schumacher, L. P. Cavalcanti, M. A. Schroer, D. I. Svergun, U. Sahin, H. Haas,Compact polyethylenimine-complexed mRNA vaccines, *Nature nanotechnology* **2025**, https://doi.org/10.1038/s41565-025-01961-w.
[67] a) S. Agarwal, Y. Zhang, S. Maji, A. Greiner,PDMAEMA based gene delivery materials, *Materials Today* **2012**, *15* (9), 388, https://doi.org/https://doi.org/10.1016/S1369-7021(12)70165-7; b) F. Schacher, M. Ulbricht, A. H. E. Müller,Self-Supporting, Double Stimuli-Responsive Porous Membranes From Polystyrene-block-poly(N,N-dimethylaminoethyl methacrylate) Diblock Copolymers, *Adv Funct Mater* **2009**, *19* (7), 1040, https://doi.org/https://doi.org/10.1002/adfm.200801457.
[68] a) J. E. Laaser, Y. Jiang, D. Sprouse, T. M. Reineke, T. P. Lodge,pH- and Ionic-Strength-Induced Contraction of Polybasic Micelles in Buffered Aqueous Solutions, *Macromolecules* **2015**, *48* (8), 2677, https://doi.org/10.1021/acs.macromol.5b00360; b) C. V. Synatschke, A. Schallon, V. Jérôme, R. Freitag, A. H. E. Müller,Influence of Polymer Architecture and Molecular Weight of Poly(2-(dimethylamino)ethyl methacrylate) Polycations on Transfection Efficiency and Cell Viability in Gene Delivery, *Biomacromolecules* **2011**, *12* (12), 4247, https://doi.org/10.1021/bm201111d.
[69] Y. Sun, J. Zhou, Q. Cheng, D. Lin, Q. Jiang, A. Dong, Z. Liang, L. Deng,Fabrication of mPEGylated graphene oxide/poly(2-dimethyl aminoethyl methacrylate) nanohybrids and their primary application

for small interfering RNA delivery, *Journal of Applied Polymer Science* **2016**, *133* (16), https://doi.org/https://doi.org/10.1002/app.43303.

[70] a) D. M. Lynn, R. Langer,Degradable Poly(β-amino esters): Synthesis, Characterization, and Self-Assembly with Plasmid DNA, *Journal of the American Chemical Society* **2000**, *122* (44), 10761, https://doi.org/10.1021/ja0015388; b) W. Cheng, D. Wu, Y. Liu,Michael Addition Polymerization of Trifunctional Amine and Acrylic Monomer: A Versatile Platform for Development of Biomaterials, *Biomacromolecules* **2016**, *17* (10), 3115, https://doi.org/10.1021/acs.biomac.6b01043.

[71] H. L. Kim, G. Saravanakumar, S. Lee, S. Jang, S. Kang, M. Park, S. Sobha, S.-H. Park, S.-M. Kim, J.-A. Lee, E. Shin, Y.-j. Kim, H.-S. Jeong, D. Kim, W. J. Kim,Poly(β-amino ester) polymer library with monomer variation for mRNA delivery, *Biomaterials* **2025**, *314*, 122896, https://doi.org/https://doi.org/10.1016/j.biomaterials.2024.122896.

[72] a) B. Chen, Q. Ren, P. Jiang, Q. Wu, Q. Shuai, Y. Yan,Combinatorial Synthesis of Alkyl Chain-Capped Poly(β-Amino Ester)s for Effective siRNA Delivery, *Macromolecular bioscience* **2024**, *24* (10), 2400168, https://doi.org/https://doi.org/10.1002/mabi.202400168; b) A. U. Bielinska, J. F. Kukowska-Latallo, J. R. Baker, Jr.,The interaction of plasmid DNA with polyamidoamine dendrimers: mechanism of complex formation and analysis of alterations induced in nuclease sensitivity and transcriptional activity of the complexed DNA, *Biochim Biophys Acta* **1997**, *1353* (2), 180, https://doi.org/10.1016/s0167-4781(97)00069-9; c) C. J. Bishop, K. L. Kozielski, J. J. Green,Exploring the role of polymer structure on intracellular nucleic acid delivery via polymeric nanoparticles, *Journal of controlled release : official journal of the Controlled Release Society* **2015**, *219*, 488, https://doi.org/10.1016/j.jconrel.2015.09.046.

[73] a) A. K. Blakney, G. Yilmaz, P. F. McKay, C. R. Becer, R. J. Shattock,One Size Does Not Fit All: The Effect of Chain Length and Charge Density of Poly(ethylene imine) Based Copolymers on Delivery of pDNA, mRNA, and RepRNA Polyplexes, *Biomacromolecules* **2018**, *19* (7), 2870, https://doi.org/10.1021/acs.biomac.8b00429; b) D. Ulkoski, M. J. Munson, M. E. Jacobson, C. R. Palmer, C. S. Carson, A. Sabirsh, J. T. Wilson, V. R. Krishnamurthy,High-Throughput Automation of Endosomolytic Polymers for mRNA Delivery, *ACS Applied Bio Materials* **2021**, https://doi.org/10.1021/acsabm.0c01463; c) E. Haladjova, V. Chrysostomou, M. Petrova, I. Ugrinova, S. Pispas, S. Rangelov,Physicochemical Properties and Biological Performance of Polymethacrylate Based Gene Delivery Vector Systems: Influence of Amino Functionalities, *Macromolecular bioscience* **2020**, e2000352, https://doi.org/10.1002/mabi.202000352.

[74] a) A. López Espinar, L. M. Mulder, M. Elkhashab, Z. Khan, M. Czarnocki-Cieciura, M. R. Aburto, S. Vucen, P. S. Kowalski,Tailoring Alkyl Side Chains of Ionizable Amino-Polyesters for Enhanced In Vivo mRNA Delivery, *ACS Applied Bio Materials* **2025**, https://doi.org/10.1021/acsabm.5c00116; b) R. Zhang, N. Zheng, Z. Song, L. Yin, J. Cheng,The effect of side-chain functionality and hydrophobicity on the gene delivery capabilities of cationic helical polypeptides, *Biomaterials* **2014**, *35* (10), 3443, https://doi.org/10.1016/j.biomaterials.2013.12.097; c) A. Biscans, J. Caiazzi, S. Davis, N. McHugh, J. Sousa, A. Khvorova,The chemical structure and phosphorothioate content of hydrophobically modified siRNAs impact extrahepatic distribution and efficacy, *Nucleic acids research* **2020**, https://doi.org/10.1093/nar/gkaa595.

[75] a) L. Wightman, R. Kircheis, V. Rössler, S. Carotta, R. Ruzicka, M. Kursa, E. Wagner,Different behavior of branched and linear polyethylenimine for gene delivery in vitro and in vivo, *The journal of gene medicine* **2001**, *3* (4), 362, https://doi.org/10.1002/jgm.187; b) M. Wojnilowicz, A. Glab, A. Bertucci, F. Caruso, F. Cavalieri,Super-resolution Imaging of Proton Sponge-Triggered Rupture of Endosomes and Cytosolic Release of Small Interfering RNA, *ACS nano* **2019**, *13* (1), 187, https://doi.org/10.1021/acsnano.8b05151; c) A. B. Cook, R. Peltier, J. Zhang, P. Gurnani, J. Tanaka, J. A. Burns, R. Dallmann, M. Hartlieb, S. Perrier,Hyperbranched poly(ethylenimine-<i>co</i>-oxazoline) by thiol–yne chemistry for non-viral gene delivery: investigating the role of polymer architecture, *Polymer Chemistry* **2019**, *10* (10), 1202, https://doi.org/10.1039/c8py01648h.

[76] T. Miyazaki, S. Uchida, S. Nagatoishi, K. Koji, T. Hong, S. Fukushima, K. Tsumoto, K. Ishihara, K. Kataoka, H. Cabral,Polymeric Nanocarriers with Controlled Chain Flexibility Boost mRNA Delivery In

Vivo through Enhanced Structural Fastening, *Advanced healthcare materials* **2020**, *9* (16), e2000538, https://doi.org/10.1002/adhm.202000538.
[77] a) Y. J. Ooi, Y. Wen, J. Zhu, X. Song, J. Li,Surface Charge Switchable Polymer/DNA Nanoparticles Responsive to Tumor Extracellular pH for Tumor-Triggered Enhanced Gene Delivery, *Biomacromolecules* **2020**, *21* (3), 1136, https://doi.org/10.1021/acs.biomac.9b01521; b) S. Mishra, P. Webster, M. E. Davis,PEGylation significantly affects cellular uptake and intracellular trafficking of non-viral gene delivery particles, *Eur J Cell Biol* **2004**, *83* (3), 97, https://doi.org/10.1078/0171-9335-00363; c) F. Richter, K. Leer, L. Martin, P. Mapfumo, J. I. Solomun, M. T. Kuchenbrod, S. Hoeppener, J. C. Brendel, A. Traeger,The impact of anionic polymers on gene delivery: how composition and assembly help evading the toxicity-efficiency dilemma, *J Nanobiotechnology* **2021**, *19* (1), 292, https://doi.org/10.1186/s12951-021-00994-2.
[78] a) D. P. Feldmann, Y. Xie, S. K. Jones, D. Yu, A. Moszczynska, O. M. Merkel,The impact of microfluidic mixing of triblock micelleplexes on in vitro / in vivo gene silencing and intracellular trafficking, *Nanotechnology* **2017**, *28* (22), 224001, https://doi.org/10.1088/1361-6528/aa6d15; b) Y. Jiang, T. P. Lodge, T. M. Reineke,Packaging pDNA by Polymeric ABC Micelles Simultaneously Achieves Colloidal Stability and Structural Control, *Journal of the American Chemical Society* **2018**, *140* (35), 11101, https://doi.org/10.1021/jacs.8b06309.
[79] a) Z. Li, L. Amaya, R. Pi, S. K. Wang, A. Ranjan, R. M. Waymouth, C. A. Blish, H. Y. Chang, P. A. Wender,Charge-altering releasable transporters enhance mRNA delivery in vitro and exhibit in vivo tropism, *Nature Communications* **2023**, *14* (1), 6983, https://doi.org/10.1038/s41467-023-42672-x; b) X. Xu, Y. Li, Q. Liang, Z. Song, F. Li, H. He, J. Wang, L. Zhu, Z. Lin, L. Yin,Efficient Gene Delivery Mediated by a Helical Polypeptide: Controlling the Membrane Activity via Multivalency and Light-Assisted Photochemical Internalization (PCI), *ACS applied materials & interfaces* **2018**, *10* (1), 256, https://doi.org/10.1021/acsami.7b15896; c) C. Jiang, Z. Qi, W. He, Z. Li, Y. Tang, Y. Wang, Y. Huang, H. Zang, H. Yang, J. Liu,Dynamically enhancing plaque targeting via a positive feedback loop using multifunctional biomimetic nanoparticles for plaque regression, *Journal of controlled release : official journal of the Controlled Release Society* **2019**, *308*, 71, https://doi.org/10.1016/j.jconrel.2019.07.007.
